# Screening of Phosphate-Solubilizing Fungi From Air and Soil in Yunnan, China: Four Novel Species in *Aspergillus*, *Gongronella*, *Penicillium*, and *Talaromyces*

**DOI:** 10.3389/fmicb.2020.585215

**Published:** 2020-10-06

**Authors:** Mingkwan Doilom, Jian-Wei Guo, Rungtiwa Phookamsak, Peter E. Mortimer, Samantha C. Karunarathna, Wei Dong, Chun-Fang Liao, Kai Yan, Dhandevi Pem, Nakarin Suwannarach, Itthayakorn Promputtha, Saisamorn Lumyong, Jian-Chu Xu

**Affiliations:** ^1^CAS Key Laboratory for Plant Diversity and Biogeography of East Asia, Kunming Institute of Botany, Chinese Academy of Sciences, Kunming, China; ^2^Honghe Innovation Center for Mountain Futures, Kunming Institute of Botany, Chinese Academy of Sciences, Kunming, China; ^3^World Agroforestry Centre, East and Central Asia, Kunming, China; ^4^Research Center of Microbial Diversity and Sustainable Utilization, Faculty of Science, Chiang Mai University, Chiang Mai, Thailand; ^5^Department of Biology, Faculty of Science, Chiang Mai University, Chiang Mai, Thailand; ^6^Institute of Plant Health, Zhongkai University of Agriculture and Engineering, Guangzhou, China; ^7^Center of Excellence in Fungal Research, Mae Fah Luang University, Chiang Rai, Thailand; ^8^Department of Entomology and Plant Pathology, Faculty of Agriculture, Chiang Mai University, Chiang Mai, Thailand; ^9^College of Resources and Environment, Yunnan Agricultural University, Kunming, China; ^10^Center of Excellence in Bioresources for Agriculture, Industry and Medicine, Department of Biology, Faculty of Science, Chiang Mai University, Chiang Mai, Thailand; ^11^Academy of Science, The Royal Society of Thailand, Bangkok, Thailand

**Keywords:** Aspergillaceae, Cunninghamellaceae, phylogeny, *Quercus* spp., taxonomy

## Abstract

Phosphate-solubilizing fungi (PSF) play an important role in increasing the bioavailability of phosphorus in soils for plants. Thirteen fungal strains, one collected from air and 12 from soil, were screened and described here in detail. These fungal strains were tested for their ability to solubilize tricalcium phosphate (TCP) on both solid and liquid Pikovskaya (PVK) media *in vitro*. The airborne fungal strain KUMCC 18-0196 (*Aspergillus hydei* sp. nov.) showed the most significant phosphate solubilizing activity on a solid PVK medium with the solubilization index (SI) (2.58 ± 0.04 cm) and the highest solubilized phosphates (1523.33 ± 47.87 μg/mL) on a liquid PVK medium. To the best of our knowledge, *A. hydei* sp. nov. is the first phosphate-solubilizing fungus reported from air. We also provide the identification especially for *Aspergillus*, *Penicillium* and *Talaromyces*, generally reported as PSF. It is important to not only screen for PSF but also identify species properly so that researchers have a clearer taxonomic picture for identifying potential taxa for future plant growth-promoting applications. Herein, *A. hydei* (section *Nigri*), *Gongronella hydei*, *Penicillium soli* (section *Lanata-Divaricata*) and *Talaromyces yunnanensis* (section *Talaromyces*) are fully described and introduced as new to science. These four new species are identified based on both morphological characteristics and multigene phylogenetic analyses, including the genealogical concordance phylogenetic species recognition method where necessary. *Penicillium austrosinense* is considered to be a synonym of *P. guaibinense.*

## Introduction

Fungi are widely distributed across terrestrial, marine, and freshwater environments (Jones et al., 2019; Phookamsak et al., 2019; Dayarathne et al., 2020; Dong et al., 2020). Fungi play important roles in both economics and ecology as sources of food and medicine that also provide decomposition services (De Silva et al., 2012a,b; Lange, 2014; Hyde et al., 2019b). Moreover, fungal research can lead to breakthroughs in microbial biotechnology and other industries (Hyde et al., 2018; Solomon et al., 2019). However, fungi can also cause diseases in humans, animals, and plants, posing numerous health challenges in need of addressing (Giacomazzi et al., 2016; Hyde et al., 2018). Given the overlooked position of studies on phosphate-solubilizing fungi (PSF), there are likely many novel fungal species still awaiting discovery.

Phosphate-solubilizing fungi (PSF) are able to enhance the solubilization of insoluble phosphate (P) compounds. They also have the capacity to mobilize and increase nutrient uptake, produce organic acids and increase the efficiency of phosphate fertilizers, such as superphosphate and rock phosphate (Jyoti et al., 2013; Li et al., 2015). Some PSF promote plant growth by secreting indole-3-acetic acid (IAA) and siderophore (Rahi et al., 2009; Zhang et al., 2018). They have enormous potential in enhancing the release of phosphorous from fertilizer (Zhang et al., 2018). PSF in soils, particularly filamentous fungi such as *Aspergillus* and *Penicillium*, have been widely investigated (Elias et al., 2016; Li Z. et al., 2016), but airborne fungi remain to be explored. In addition, previous identifications of PSF (e.g., *Aspergillus*, *Penicillium*, and *Talaromyces*) have been based on morphology, nucleotide similarities in MegaBLAST searches of NCBI’s GenBank nucleotide database and/or phylogenetic analysis of ITS sequence data (e.g., Vyas et al., 2007; Nelofer et al., 2015; Qiao et al., 2019).

In this paper, 13 fungal strains from air and soil samples were screened for their ability to solubilize phosphate. Known species are identified on the basis of phylogenetic analyses. New species are introduced based on morphological characteristics and phylogenetic analyses. This study provides new scientific insights into novel PSF isolated from the air. In addition, new fungal taxa isolated from the soil of cultivated oak seedlings (*Quercus rubra*) in Honghe County, Yunnan Province, China are introduced.

## Materials and Methods

### Collection and Isolation of Fungi From Air Using the Settle Plate Method

Five Petri plates (9 cm diam.) with opened lids containing PVK agar with 5% tricalcium phosphate [TCP, Ca_3_(PO_4_)_2_] were placed under a *Quercus variabilis* tree in Kunming City, Yunnan Province, China and left for 20 min. *Quercus variabilis* is a selected host in this study as it is a native oak in China. Lids were then closed over the plates and carefully transferred to the laboratory where they were incubated for 2 days at room temperature. The fungal colony with the widest clear zone around the colony on PVK agar was selected and transferred to fresh potato dextrose agar (PDA) to obtain a phosphate-solubilizing fungus.

### Collection and Isolation of Fungi From Soil Using Soil Dilutions

Twenty grams of rhizospheric soil samples (10 cm depth) were collected from *Quercus rubra* in Honghe County, Yunnan Province, China. *Quercus rubra* is also a selected host herein as it is the most valuable timber species in red oak group. Soil samples were separated from roots, stored in ziplock bags and carefully transferred to the laboratory. One gram of each soil sample was dissolved into 9 mL of sterilized distilled water in 15 mL test tubes and mixed well. The serial soil dilutions were made for 10^–4^, 10^–5^, 10^–6^. Soil dilutions were used to isolate fungi from soil samples. Half a milliliter (0.5 mL) of each dilution was drawn using a micropipette and placed onto a PDA plate supplemented with 100 mg/mL streptomycin and spread using a sterile L-shaped spreader. The Petri plates were then inoculated for 2–5 days at room temperature. Fungal colonies were purified by transferring a single hyphal tip to PDA. Pure cultures of each fungal isolate were preserved on PDA agar slants in 2 mL vial tubes at 4°C for further screening.

### Determination of Solubilization Index on PVK Agar

Thirteen fungal isolates obtained from pure cultures were preliminarily screened for their potential to solubilize TCP as insoluble inorganic phosphate sources on PVK agar. One liter (1 L) of PVK agar contained the following (g/L): 0.5 g (NH_4_)_2_SO_4_, 0.1 g MgSO_4_⋅7H_2_O, 0.02 g NaCl, 0.02 g KCl, 0.003 g FeSO_4_⋅7H_2_O, 0.003 g MnSO_4_⋅H_2_O, 5 g Ca_3_ (PO_4_)_2_, 10 g glucose, 0.5 g yeast extract, 15 g agar, and 1000 mL distilled water (Pikovskaya, 1948). The medium was autoclaved at 121°C for 15 min. Sterilized PVK agar was poured into sterilized Petri plates. Fungal mycelium plugs of each isolate grown on PDA at 28°C for 7 days were cut from the edges of each actively growing colony using a sterile cork borer (5 mm^3^). Fungal mycelium plugs were then placed on Petri plates containing PVK agar supplemented with 0.5% TCP for 7 days at 28°C. Plugs of sterile PDA were used as controls. Three replicates were tested for each fungal isolate. The diameter of clear zones around the colony of each isolate was measured after the 1^st^, 3^rd^, 5^th^, and 7^th^ day of incubation. The phosphate solubilization index was calculated according to the formula below (Premono et al., 1996).

$$\mathrm{Solubilization}\,\mathrm{Index}\,\left(\mathrm{SI}\right)=\frac{\mathrm{colony}\,\mathrm{diameter}+\mathrm{clearing}\,\mathrm{zone}\,\mathrm{diameter}}{\mathrm{colony}\,\mathrm{diameter}}$$

### Phosphate Solubilization Efficiency of Fungal Isolates to Tricalcium Phosphate in PVK Broth

The phosphate solubilization activity test was carried out in a 150 mL conical flask with 100 mL PVK broth supplemented with 0.5% TCP. The initial pH of the medium was adjusted to 7.0 before sterilization. The preparation of spore suspensions from each fungal isolate was done according to the method described in Elias et al. (2016). Ten milliliter (10 mL) spore suspensions of each fungal culture (10^7^ spores/mL) were inoculated in the sterilized PVK broth of each conical flask. Ten milliliters (10 mL) of sterile distilled water in the sterilized PVK broth was treated as the control. Three replicates were maintained for each fungal test. The cultures were incubated on a rotary shaker (Sanco, India) at 28°C at 130 rpm for 7 days. Aliquots of 1.5 mL culture supernatant were aseptically collected from each conical flask on the 1^st^, 3^rd^, 5^th^, and 7^th^ day. The samples were centrifuged (Sigma, Germany) at 12,000 rpm for 2 min to discard any suspended solids and mycelial fragments. Supernatants (0.1 mL) of each culture were then taken out to estimate the amount of phosphorus released from TCP of fungal strains. The available soluble phosphate in culture supernatants was estimated using the molybdenum blue method under 700 nm (Ryan et al., 2001). The pH of the culture supernatant in PVK broth was also measured with a digital pH meter FE 28 equipped with a glass electrode (Shanghai, Co., Ltd.).

### Statistical Analysis

Data from different treatments were calculated and statistically analyzed with a one-way analysis of variance (ANOVA) using the Statistical Package for the Social Sciences (SPSS) for Windows version 22 (SPSS, Inc., Chicago, IL, United States). Duncan’s multiple range tests were used to determine significant differences at *p* < 0.05 between the mean values.

### Morphological Studies

The fungi were identified using morphological characteristics and phylogenetic analyses. New taxa are introduced with full descriptions. Fungal strains grown on PDA and MEA were mounted in lactic acid for microscopic examination. Morphological observations and photomicrographs were made with a Nikon ECLIPSE 80i compound microscope fitted with a Canon 600D digital camera. Morphological characteristic measurements were made with the Tarosoft (R) Image Frame Work. Images used for figures were processed with Adobe Photoshop CS6 (Adobe Systems, United States).

Type herbarium materials were deposited in the herbarium of Mae Fah Luang University (MFLU) Chiang Rai, Thailand. Ex-type living cultures were deposited in the Kunming Institute of Botany Culture Collection (KMUCC) Yunnan, China.

### DNA Extraction, PCR Amplification, and Sequencing

The DNA was extracted from the mycelium grown on PDA at 25°C for 7 days using a Biospin Fungus Genomic DNA Extraction Kit–BSC14S1 (BioFlux^®^, China) following the manufacturer’s protocol. The 5.8S nrRNA gene with the two flanking internal transcribed spacers (ITS) regions were first amplified and sequenced using the primers ITS1/ITS4 (White et al., 1990) for all fungal strains. They were preliminarily identified to genera level as *Aspergillus*, *Penicillium*, *Talaromyces*, *Gongronella*, and *Fusarium* based on the BLAST searches of ITS in the nucleotide database of GenBank^1^. Partial calmodulin gene (*CaM*) using primers CMD5/CMD6 (Hong et al., 2005), partial beta-tubulin gene (*BenA*) using primers Bt2a/Bt2b (Glass and Donaldson, 1995) and partial RNA polymerase II second largest subunit (*RPB2*) using primers 5F/7CR (Liu et al., 1999) were then amplified and sequenced for strains of *Aspergillus*, *Penicillium*, and *Talaromyces*. The LSU gene region was amplified and sequenced using the primers LR0R and LR5 (Vilgalys and Hester, 1990; Rehner and Samuels, 1994) for the strains of *Gongronella*. Translation elongation factor 1-alpha (TEF1-α) was amplified and sequenced using primers TEF1/TEF2 (O’Donnell et al., 1998) for the strain of *Fusarium* but was unsuccessful even after several efforts.

The PCR amplifications were conducted in 25 μL final volumes which consisted of 12.5 μL of 2 × Power Taq PCR MasterMix (a premix and ready to use solution, including 0.1 Units/μL Taq DNA Polymerase, 500 μM dNTP Mixture each (dATP, dCTP, dGTP, dTTP), 20 mM Tris-HCl pH 8.3, 100 mM KCl, 3 mM MgCl_2_, stabilizer and enhancer), 1 μL of each primer (10 μM), 1 μL genomic DNA extract and 9.5 μL distilled-deionized water. Thermocycling was completed on a 2720 Thermal Cycler (Applied Biosystems, Foster City, CA, United States) under the following conditions: initial denaturation of 5 min at 95°C, followed by 35 cycles of 1 min at 95°C, 2 min at 52°C, 90 s at 72°C and a final extension period of 10 min at 72°C for ITS and *RPB2*. The annealing temperature was changed to 55°C for *CaM*, LSU, TEF, and *BenA*. The amplified PCR products were purified and sequenced by BGI Tech Solutions, Co. Ltd. (BGITech), China.

### Phylogenetic Analyses

Consensus sequences were generated using Geneious^®^ R7 (Biomatters, Ltd., New Zealand) and BioEdit (Hall, 1999). Sequences were BLAST searched in the nucleotide database of GenBank^1^ to examine their closely related taxa. The reference sequences and outgroups were selected from recent relevant literature and GenBank (Supplementary Table S1). The datasets of genomic regions were aligned individually using the MAFFT online version 7.450 server^2^. Phylogenetic trees were inferred with maximum parsimony (MP), maximum likelihood (ML) and Bayesian inference (BI), according to the details described in Wanasinghe et al. (2018).

### Genealogical Concordance Phylogenetic Species Recognition (GR)

The new *Aspergillus* taxon and closely related species were analyzed separately using the GCPSR model. The pairwise homoplasy index (PHI) was determined using SplitsTree v. 4.15.1 (Huson and Bryant, 2006^3^) as described by Taylor et al. (2000) and Quaedvlieg et al. (2014) to determine the recombination level within phylogenetically closely related species. A PHI value below 0.05 (Φw < 0.05) indicates the presence of significant recombination in the dataset. Split graphs were constructed to ease visualization of the relationship between closely related species. The relationships between this new taxon and closely related species were visualized by constructing splits graphs using both the LogDet transformation and splits decomposition options.

## Results

### Formation of Clear Zones and Phosphate Solubilization Index (SI) on PVK Agar

**FIGURE 1:**
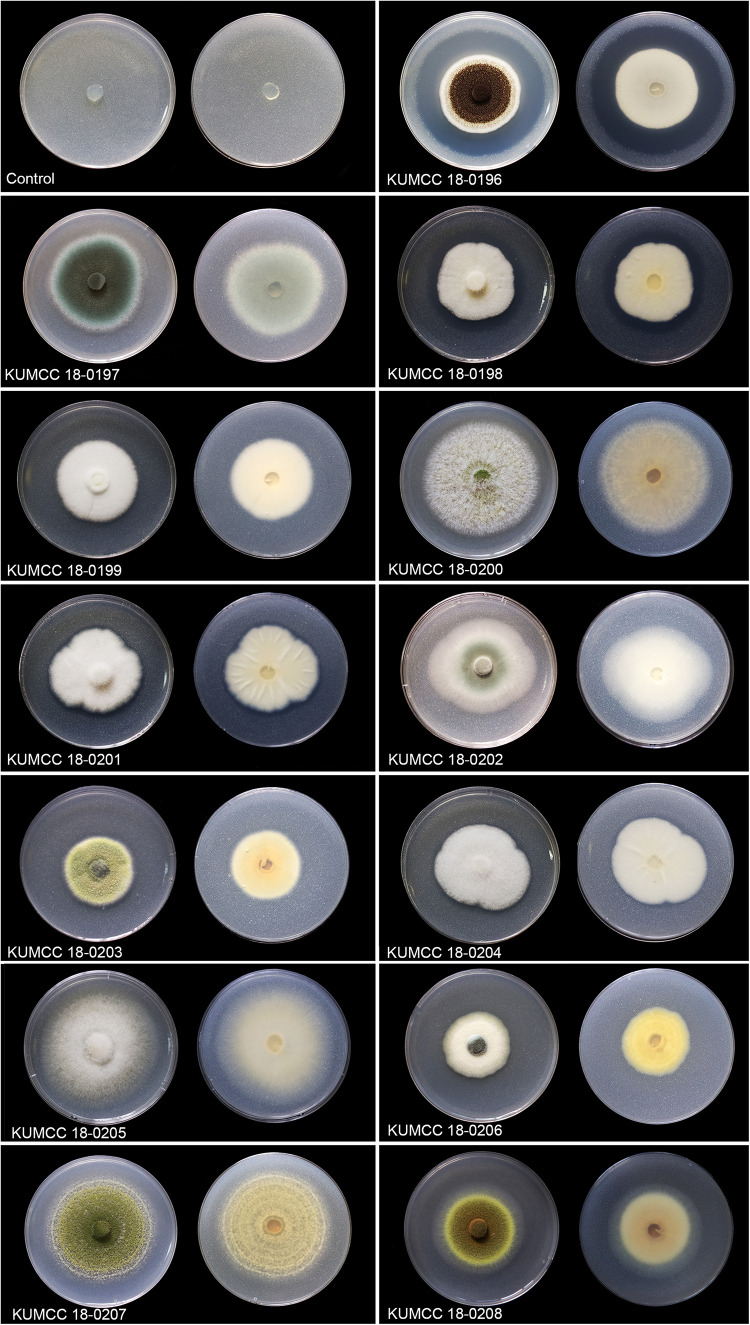
Colony features and clear zones formation for TCP solubilization on PVK agar after 7 days Except isolate KUMCC 18-0196 after 5 days (left = above view, right = below).

The clear zone became visible in the isolates KUMCC 18-0196 and KUMCC 18-0206 on the 1^st^ day. The clear zone of the other fungal isolates became visible on the 3^rd^ day and did not show the clear zone around colonies after the 7^th^ day but solubilized TCP by removing white spots on PVK agar (Figure 1). The isolate KUMCC 18-0196 showed the widest clear zone around the fungal colony (Figure 1).

**FIGURE 2:**
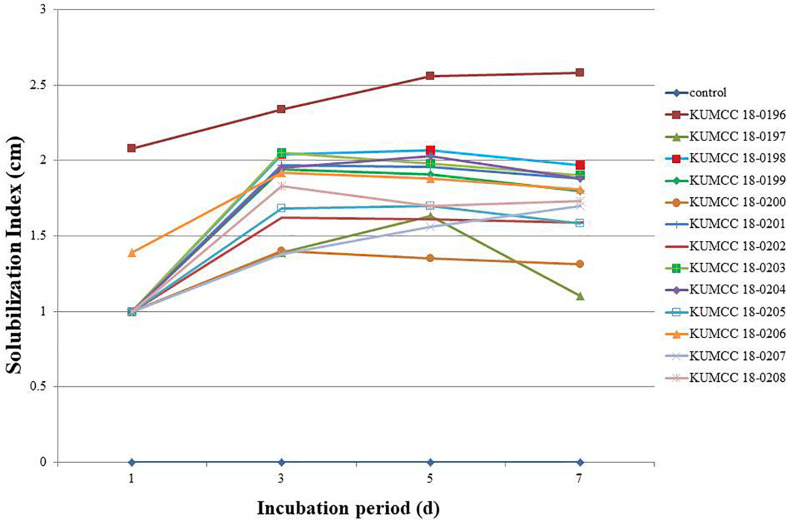
Solubilization index (SI) of 13 fungal strains and control on PVK agar containing TCP after the 1^st^, 3^rd^, 5^th^, and 7^th^ day of incubation (each value represents the mean of the three replicates).

The phosphate solubilization index began increasing in isolates KUMCC 18-0196 and KUMCC 18-0206 on the 1^st^ day, while in other fungal isolates it began increasing on the 3^rd^ day (Figure 2). The highest phosphate solubilization index was seen in KUMCC 18-0196 (SI = 2.58 ± 0.04 cm) compared with other fungal strains and the control on the 7^th^ day, followed by KUMCC 18-0198 with SI = 1.97 ± 0.15 cm and KUMCC 18-0203 with SI = 1.90 ± 0.01 cm (Supplementary Table S2). It was observed that isolate KUMCC 18-0197 had the smallest SI = 1.10 ± 0.17 cm. The phosphate solubilization of isolate KUMCC 18-0196 was significantly different (*p* < 0.05) from other fungal strains (Supplementary Table S2).

### Phosphate Solubilization and pH in PVK Broth

**FIGURE 3:**
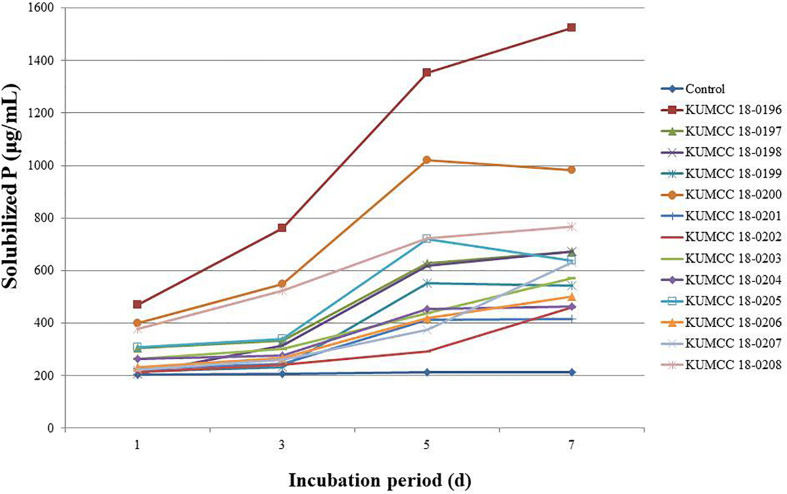
Solubilized P concentrations of 13 fungal strains and control in PVK broth containing TCP after the 1^st^, 3^rd^, 5^th^, and 7^th^ day of incubation (each value represents the mean of the S3). pH values of PVK broth containing TCP inoculated with each 13 fungal isolate and control after the 1^st^, 3^rd^, 5^th^, and 7^th^ day of incubation (each value represents the mean of the three replicates).

All fungal strains released phosphate on the 1^st^ day and gradually increased when compared with the control. Isolates KUMCC 18-0199, KUMCC 18-0200 and KUMCC 18-0205 later decreased on the 7^th^ day of incubation, while isolates KUMCC 18-0196, KUMCC 18-0197, KUMCC 18-0198, KUMCC 18-0201, KUMCC 18-0202, KUMCC 18-0203, KUMCC 18-0204, KUMCC 18-0206, KUMCC 18-0207, and KUMCC 18-0208 increased (Figure 3). Isolate KUMCC 18-0196 released solubilized phosphate (1523.33 ± 47.87 μg/mL) with significant difference (*p* < 0.05) compared to other fungal strains (Supplementary Table S2). The smallest amount of solubilized phosphate was detected in isolate KUMCC 18-0201 (417.33 ± 22.00 μg/mL) (Supplementary Table S2).

**FIGURE 4:**
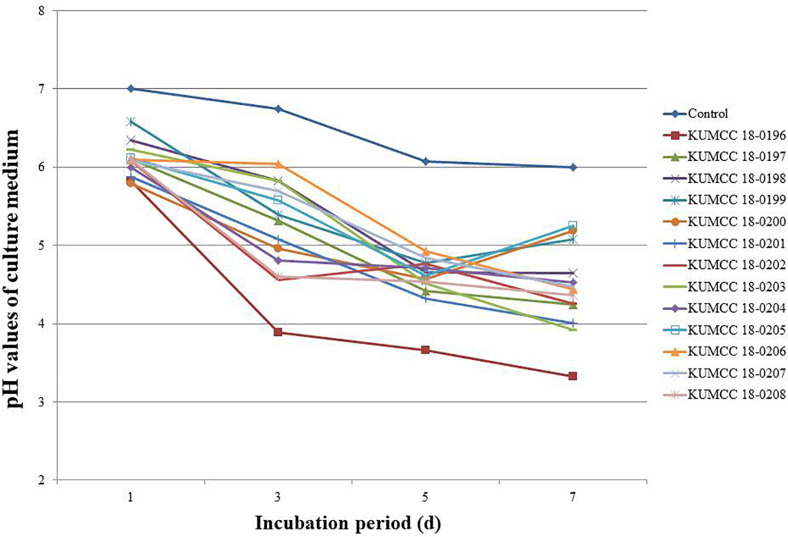
pH values of PVK broth containing TCP inoculated with each 13 fungal isolate and control after the 1^st^, 3^rd^, 5^th^, and 7^th^ day of incubation (each value represents the mean of the three replicates).

The pH values of all 13 fungal isolates decreased from 7.01 after the 1^st^ day of incubation. The pH values continued to decrease after the 3^rd^ day and increased or decreased to variable levels in the PVK broth containing TCP during the 5^th^ and 7^th^ days of incubation, depending on the fungal isolates (Figure 4). The isolate KUMCC 18-0196 showed the highest decrease in pH from an initial pH of 7.01 to 3.33 ± 0.01 on the 7^th^ day of incubation, which was significantly different compared to the control and other fungal strains (Supplementary Table S2).

### Taxonomic and Phylogenic Studies

In total, 13 fungal strains screened for TCP solubilization on both solid and liquid PVK media are listed in Supplementary Table S3. Five strains are introduced as new species viz. *Aspergillus hydei*, *Gongronella hydei*, *Penicillium soli* and *Talaromyces yunnanensis*, based on morphological characteristics and phylogenetic analyses. *Penicillium guaibinense* collected from soil in China is also illustrated and described. Their morphological characteristics and phylogenetic analyses are provided below.

#### *Aspergillus* Section *Nigri* W. Gams, M. Chr., Onions, Pitt, and Samson

*Type*: *Aspergillus niger* Tiegh.

*Notes*: *Aspergillus* section *Nigri* is the black aspergilli. This section includes species with smooth conidiophores and hyaline or pigmented below the vesicle, globose, subglobose to pyriform vesicles, typically radiate conidial heads, or divergent columns in some species, and black conidia (Gams et al., 1986). They have been isolated from various habitats, such as plants, contaminated materials, indoor air environments and soil samples (Sørensen et al., 2011; Visagie et al., 2014a; Fungaro et al., 2017; Serra et al., 2017; this study). Many strains of *Aspergillus niger* are used in biotechnology for the production of various metabolites, such as in antibiotics, organic acids, bioenergy or as microbial fermentation in food (Hasegawa et al., 2007; Show et al., 2015; Chuppa-Tostain et al., 2018). They have also been reported as the pathogen responsible for invasive pulmonary aspergillosis (Walsh et al., 2007) and other plant pathogens (Gautam et al., 2011; Sharma, 2012; Guo et al., 2017). Black aspergilli are hard to differentiate solely by morphological characteristics due to high species variation. We introduce the new species *A. hydei* (KUMCC 18-0196) from air based on morphological characteristics and multi-loci phylogenetic analyses, including the GCPSR method.

#### *Aspergillus hydei* Doilom, sp. nov., Figure 5

*Index Fungorum number*: IF 557860; *Facesoffungi number*: FoF 08837

**FIGURE 5:**
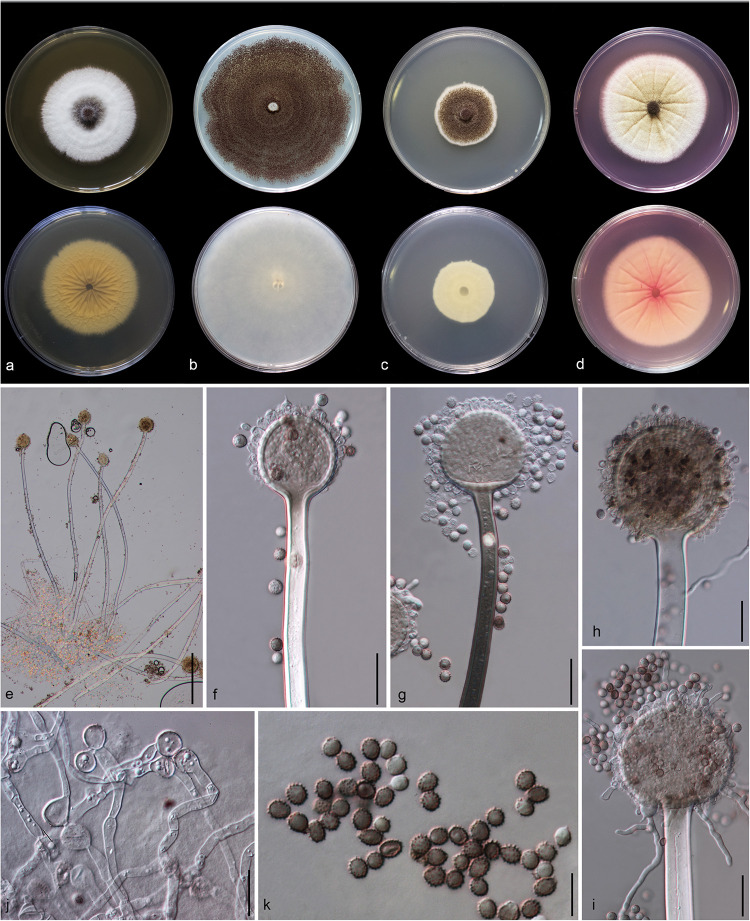
*Aspergillus hydei* (MFLU 20-0430, holotype). **(a–d)** Colonies on MEA, CYA, PDA and Rosebengal at 25°C, respectively. **(e)** Conidiophores. **(f–i)** Close-up of conidiophores, vesicle, phialides, and conidia (**f,g:** exhibiting colors of polarized light under DIC illumination). **(j)** Chlamydospores. **(k)** Conidia. Scale bars: **(e)** = 200 μm, **(f–j)** = 20 μm, **(k)** = 10 μm.

*Etymology*: Named in honor of Kevin D. Hyde for his excellent contributions to mycology.

*Holotype*: MFLU 20-0430

*Macromorphology*: *Colonies on MEA* reaching 50–55 mm diam. after 7 days in the dark at 25°C, circular to filamentous, undulate to filamentous, flat or effuse to raise, dense, compact white basal felt covered by a dense layer of dark brown to black conidial heads in the center, white at the edge from above; yellow or creamy, wrinkled and cracked from below. *Colonies on Czapeck Yeast Agar* (CYA) reaching 75–80 mm diam. after 7 days in the dark at 25°C, irregular in shape, curled to undulate, flat or effuse, compact white basal felt covered by a dense layer of black conidial heads, with concentric rings from above; white from below. *Colonies on PDA* reaching 35–40 mm diam. after 7 days in the dark at 25°C, filamentous shape, undulate to filamentous, flat or effuse to raise, dense, compact white basal felt covered by a dense layer of dark brown to black conidial heads, white at the edge from above; white or creamy from below. *Colonies on WA* reaching 50–55 mm diam. after 7 days in the dark at 25°C, slightly circular, edge entire to undulate, flat, loose, white with black conidial heads at the center (mycelium plug) from above; white from below. *Colonies on Rosebengal* reaching 60–65 mm diam. after 7 days in the dark at 25°C, slightly circular, edge entire to undulate, flat, loose, compact white basal felt covered by a lose layer of cream conidial heads with gray conidial heads at the center (mycelium plug), with wrinkled from the center to the edge, white at the edge from above; white to cream from below.

*Micromorphology*: *Conidial heads* globose, dark brown, becoming black, radiate, split into several loose columns with age, uniseriate. *Conidiophores* up to 1200 μm long, 14–18 μm wide, smooth-walled, hyaline or pigmented, aseptate, unbranched, straight to curved. *Vesicles* 32–64 μm wide ($\bar{x}$ = 56.3 × 52.2 μm, *n* = 15), globose to subglobose, uniseriate, fertile over entire surface, without metula; phialides 4–8 × 3.5–9 μm ($\bar{x}$ = 5.3 × 4.9 μm, *n* = 20), hyaline, flask-shaped, ampulliform. *Conidia* 4.3–6 × 4–6.2 μm ($\bar{x}$ = 4.7 × 4.8 μm, *n* = 20), globose to subglobose, sometime ellipsoidal, initially hyaline, becoming reddish brown to black, with coarsely roughened to echinulate surface, rough-walled.

*Material examined*: CHINA, Yunnan Province, Kunming City, from air under *Quercus variabilis*, 20 March, 2018, M. Doilom (MFLU 20-0430, **holotype**); ex-type living culture (KUMCC 18-0196).

**FIGURE 6:**
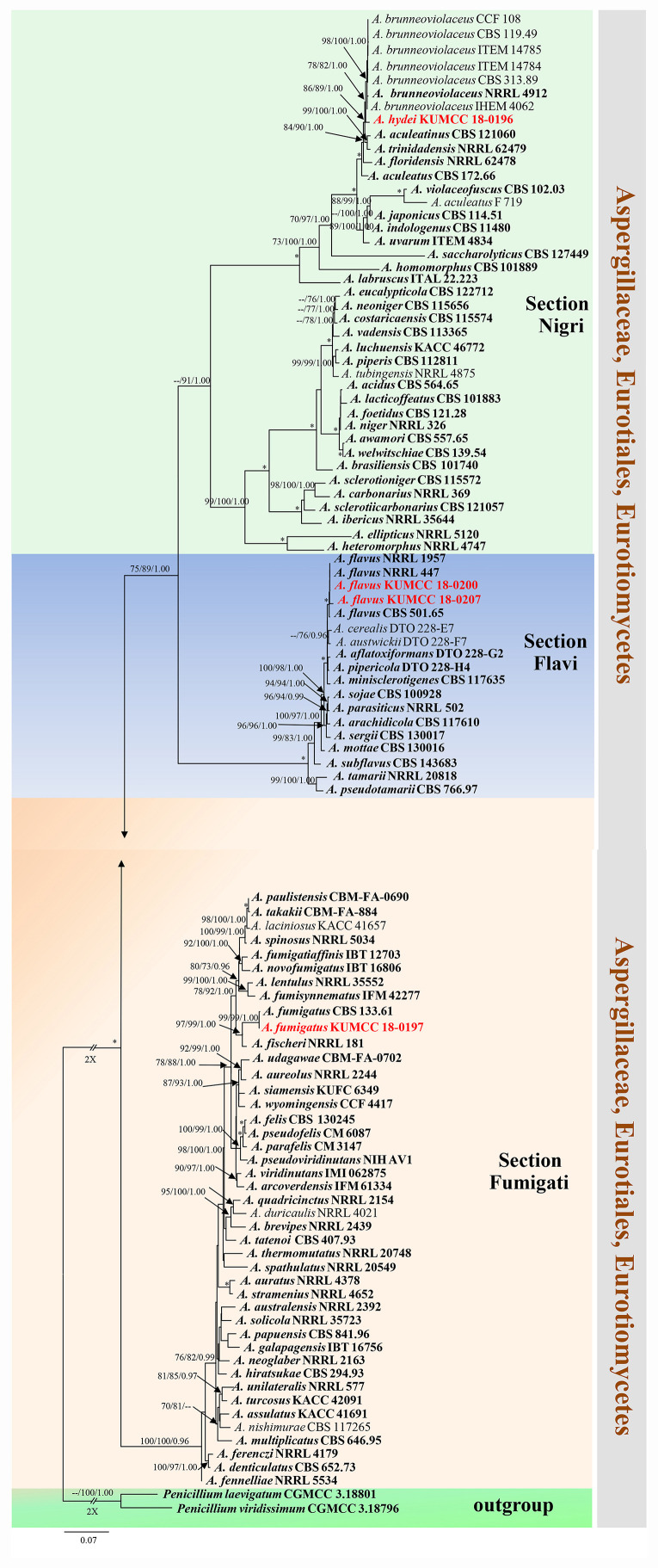
Phylogram generated from maximum likelihood analysis based on combined *CaM*, *BenA*, ITS and *RPB2* sequence data. One hundred and three sequences are included in the combined analysis, which consisted of 2805 characters including alignment gaps. The tree is rooted to *Penicillium viridissimum* (CGMCC 3.18796) and *P. laevigatum* (CGMCC 3.18801). The MP analysis for the combined dataset had 1199 parsimony informative, 1432 constant, 174 parsimony uninformative and yielded 356 most parsimonious trees. The best-fit model HKY + I + G was selected for *CaM*, *BenA*, GTR + I + G for ITS and *RPB2* in BI analysis. Maximum parsimony and maximum likelihood bootstrap values ≥ 70% and Bayesian posterior probabilities ≥ 0.90 (MPBS/MLBS/BYPP) are indicated at the nodes. Branches with 100% MPBS, 100% MLBS and 1.00 BYPP values are indicated as an asterisk (^∗^). The ex-type strains are bolded black, and the new isolates are in red.

**FIGURE 7:**
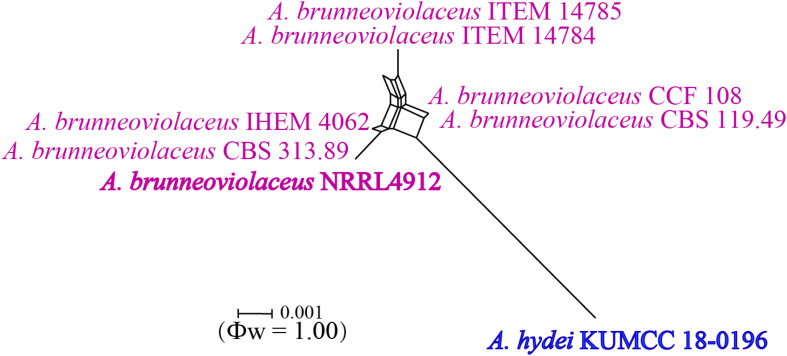
Split graph showing the results of the pairwise homoplasy index (PHI) test of the combined *CaM*, *BenA*, ITS and *RPB2* sequence data between *Aspergillus brunneoviolaceus* and *A. hydei* using LogDet transformation and splits decomposition. PHI test result (Φw) < 0.05 indicates significant recombination within the dataset. Ex-type strains are indicated in bold.

*Notes*: Colony diameter after 7 days of incubation at 25°C on CYA of *A. hydei* is obviously smaller than *A. brunneoviolaceus* (75–80 vs. > 85 mm) (Jurjević et al., 2012). *A. hydei* (MFLUCC 18-0196) had a close phylogenetic affinity with *A. brunneoviolaceus* based on individual ITS and *CaM* sequence data (Supplementary Figures S1, S2). However, *A. hydei* formed an independent lineage to the ex-type (NRRL 4912) and other strains of *A. brunneoviolaceus* based on combined *CaM*, *BenA*, ITS and *RPB2* sequence data with significant support (78% MP, 82% ML and 1.00 PP) (Figure 6). *A. hydei* also had close phylogenetic affinity with *A. aculeatinus*, *A. aculeatus*, *A. floridensis*, and *A. trinidadensis* (Supplementary Figures S3, S4) but formed a distinct lineage from these four species (Figure 6). The GCPSR test was applied to a combined dataset including *A. hydei* and its closely related *A. brunneoviolaceus*, containing eight isolates. The result revealed that there was no significant recombination between *A. brunneoviolaceus* and *A. hydei* (Φw = 1.00) (Figure 7). Thus, *A. hydei* can be recognized as a new species.

#### *Gongronella* Ribaldi

*Type*: *Gongronella urceolifera* Ribaldi

*Notes*: Species of *Gongronella* have mainly been isolated from soil (Akone et al., 2014; Adamčík et al., 2015; Ariyawansa et al., 2015). Morphological characteristics of *Gongronella* species are diverse in shape, making it difficult to clearly delineate the species belonging to this genus. In addition, *Gongronella* species have been described from different media (Hesseltine and Ellis, 1961, 1964; Adamčík et al., 2015; Ariyawansa et al., 2015; Zhang et al., 2019). However, *Gongronella* species can be separated by phylogenetic analysis based on ITS sequences as shown in Li G. J. et al. (2016) and Tibpromma et al. (2017) and this study. Multi-locus gene regions would well-resolve to determine the species. The genus comprises nine epithets in Index Fungorum (2020). *G. hydei* is introduced as a new species herein.

#### *Gongronella hydei* Doilom, sp. nov., Figure 8

*Index Fungorum number*: IF 557861; *Facesoffungi number*: FoF 08838

**FIGURE 8:**
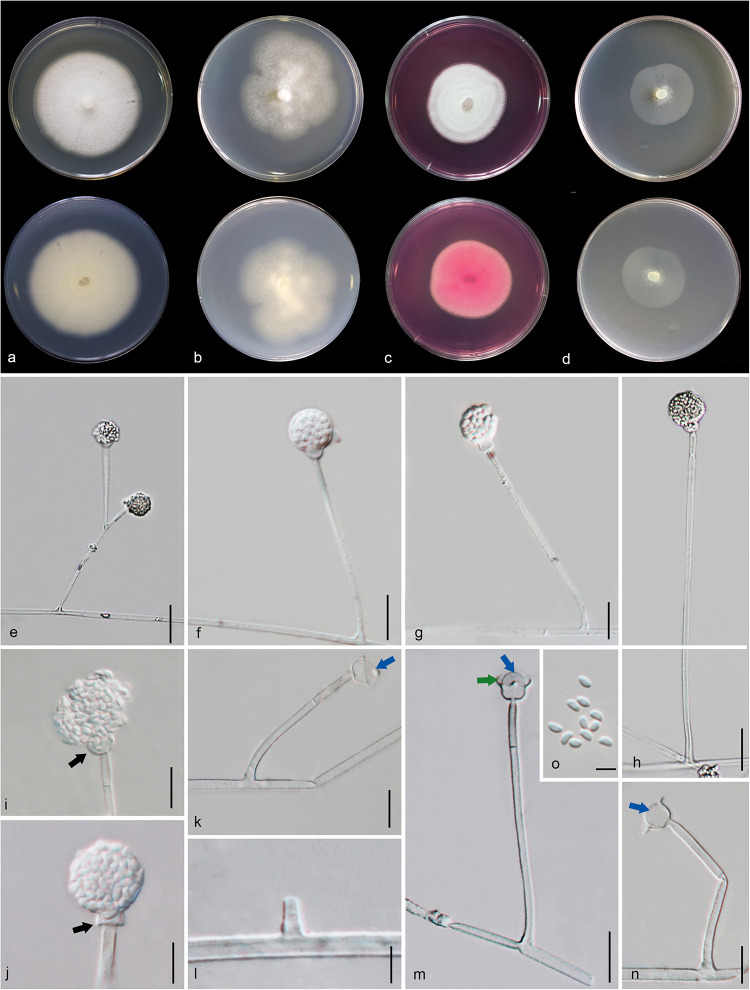
*Gongronella hydei* (MFLU 20-0431, holotype). **(a–d)** Colonies on PDA, CYA, rosebengal and WA at 25°C, respectively. **(e)** Branched sporangiophore with sporangia. **(f–h)** Sporangiophores with sporangia and apophyses. **(i,j)** Aggregated sporangiospores in sporangia with variously shaped apophysis (black arrows). **(k,m,n)** Septate sporangiophores with apophyses, columella (blue arrows) and collarette (green arrow). **(l)** Developed sporangiophore from hypha. **(o)** Sporangiospores. Scale bars: **(e,h)** = 20 μm, **(f,g,i–n)** = 10 μm, **(o)** = 5 μm.

*Etymology*: Named in honor of Kevin D. Hyde for his excellent contributions to mycology.

*Holotype*: MFLU 20-0431

*Macromorphology*: *Colonies on PDA* reaching 60–65 mm diam. after 7 days in the dark at 25°C, circular, edge entire, flat or effuse, to raise, dense, white from above and below. *Colonies on CYA* reaching 55–65 mm diam. after 7 days in the dark at 25°C, irregular in shape, curled to undulate, flat or effuse, medium dense, white from above and below. *Colonies on Rosebengal* reaching 45–47 mm diam. after 7 days in the dark at 25°C, slightly circular, edge entire to undulate, filamentous, flat, dense, cottony, concentric ring, white from above and below. *Colonies on WA* reaching 30–35 mm diam. after 7 days in the dark at 25°C, irregular in shape, curled, flat, loose, with obviously white filamentous around the center (mycelium plug) from above; white from below.

*Micromorphology*: *Rhizoids* absent. *Sporangiophores* on PDA up to 120 μm long, 1.6–3.2 μm wide, erect, arising from hyphae, straight or flexuous, smooth- to minutely rough-walled, hyaline, unbranched, occasionally branched, 1–2-septate, mostly 1-septate below the apophysis. *Apophyses* variable in shape, cuboid-shaped with truncate at the base: 2.5–3.9 × 3.5–5.1 μm; cup-shaped with rounded at the base: 2.7–6.2 × 3.8–7.8 μm; cup-shaped with truncate at the base: 3.7–7.3 × 3.8–7.3 μm, hyaline, smooth-walled. *Sporangia* 10.5–18.8 × 10–17.5 μm, hyaline, globose to subglobose, thin-walled, occasionally thick-walled, smooth-walled, multi-spored, with an apophyses. *Columellae* 1.7–4.7 × 2.2–6.3 μm, hemispherical with a collarette, smooth-walled, sometime tiny, almost inconspicuous, mostly with an evident collar. *Sporangiospores* variable in shape, reniform: 2.4–3.8 × 1.5–2.3 μm, ellipsoidal to fusiform: 2.6–3.4 × 1.8–3.4 μm, hyaline, smooth-walled, aseptate, guttulate. *Chlamydospores* present in the aerial mycelium, globose. *Giant cells* up to 25 μm diam., hyaline, globose, guttulate. *Zygosporangia* not observed.

*Material examined*: CHINA, Yunnan Province, Honghe County, from rhizosphere soil of *Quercus rubra*, 16 April 2018, M. Doilom (MFLU 20-0431, **holotype**); ex-type living culture (KUMCC 18-0198), ibid., living culture KUMCC 18-0204.

**FIGURE 9:**
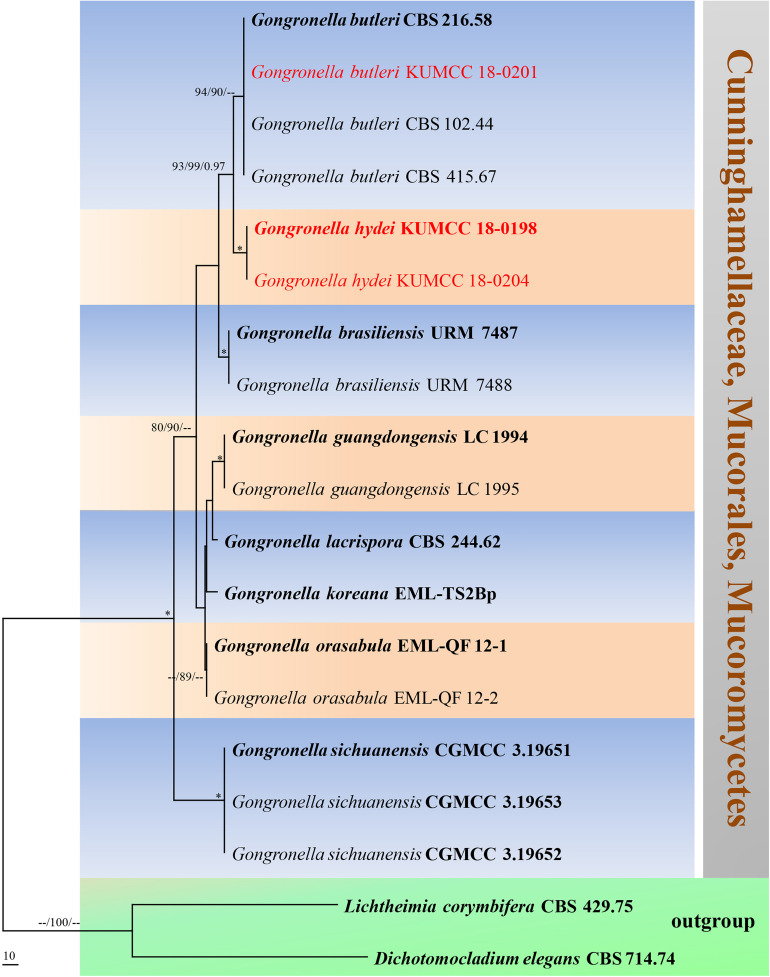
Phylogram generated from maximum parsimony analysis based on ITS sequence data. Nineteen sequences are included in the ITS analysis, which consisted of 772 characters including alignment gaps. The tree is rooted to *Lichtheimia corymbifera* (CBS 429.75) and *Dichotomocladium elegans* (CBS 714.74). The MP analysis for the ITS dataset had 187 parsimony informative, 315 constant, 270 parsimony uninformative characters and yielded two most parsimonious trees. The best-fit model GTR + G was selected for ITS. Maximum parsimony and maximum likelihood bootstrap values ≥ 70% and Bayesian posterior probabilities ≥ 0.90 (MPBS/MLBS/BYPP) are given at the nodes. The ex-type strains are bolded, and the new isolates are in red. Branches with 100% MPBS, 100% MLBS and 1.00 BYPP values are indicated as an asterisk (^∗^).

*Notes*: The novel *G. hydei* lacks rhizoids, which are present in *G. butleri* (Hesseltine and Ellis, 1964). However, *G. hydei* was observed on PDA, while *G. butleri* observed on SMA (synthetic mucor agar). Phylogenetic analysis of ITS sequences confirmed that our isolates (KUMCC 18-0198 and KUMCC 18-0204) grouped clearly distinct from other known species (Figure 9). An isolate KUMCC 18-0198 grouped with KUMCC 18-0204 (100% MP, 100% ML and 1.00 PP) but separated from other *G. butleri* strains with 93% MP, 99% ML and 0.97 PP (Figure 9). Thus, we introduce *G. hydei* as a new species.

#### *Penicillium* Section *Lanata-Divaricata* Raper and Thom ex Pitt

*Type*: *Penicillium janthinellum* Biourge

*Notes*: Species of *Penicillium* in the section *Lanata-Divaricata* are characterized by divaricate conidiophores and broadly scattering colonies (Houbraken and Samson, 2011). Members of *Penicillium* are widespread in food products, soil, plant-decaying materials, animals and indoor environments (Visagie et al., 2016; Diao et al., 2018; Heo et al., 2019). Many recent studies reported *Penicillium* strains belonging to the section *Lanata-Divaricata* (Visagie et al., 2016; Lorenzini et al., 2019; Hyde et al., 2019a; Guevara-Suarez et al., 2020). Delimiting species in this section based only on morphological characteristics is difficult; accordingly, ITS, *BenA*, *CaM* and *RPB2* are used to delimit species boundaries (Visagie et al., 2016). *P. soli* is introduced as a new species herein.

#### *Penicillium guaibinense* J. P. Andrade, C. N. Figueiredo, R. P. Nascimento, P. A. S. Marbach, and J. T. De Souza, in Crous et al., Persoonia 41: 389 (2018), Figures 10, 11

= *Penicillium austrosinense* L. Cai, Houbraken and X. Z. Jiang, in Diao, Chen, Jiang, Houbraken, Barbosa, Cai and Wu, Cladistics: 10.1111/cla.12365, 12 (2018)

**FIGURE 10:**
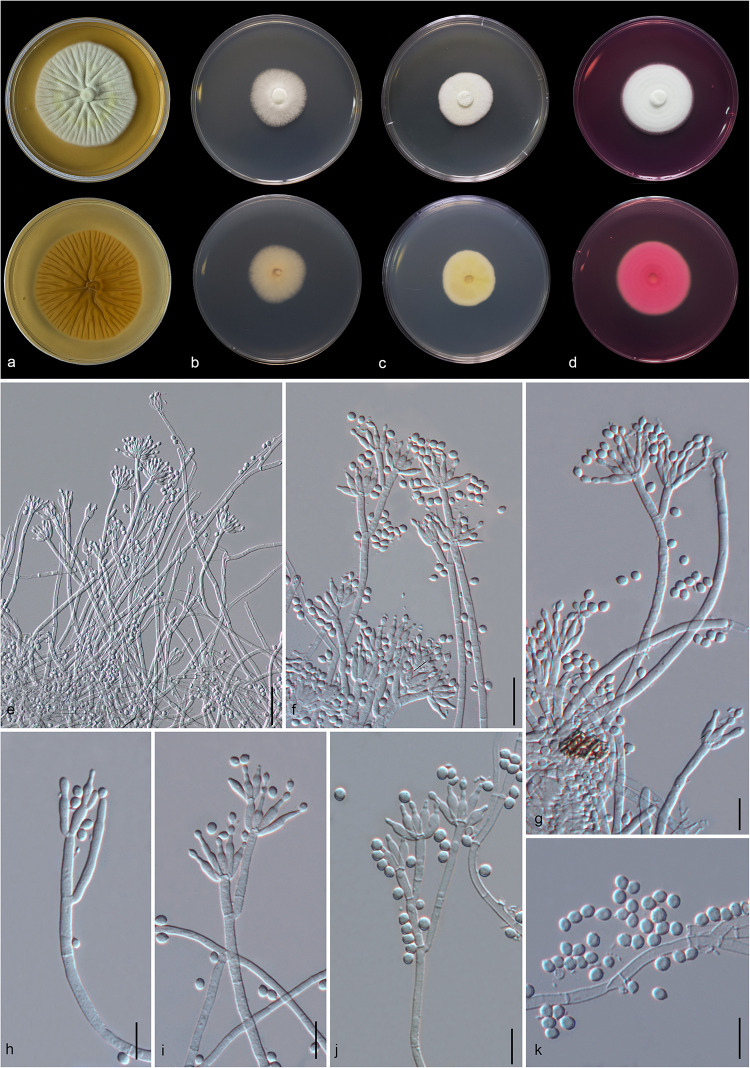
*Penicillium guaibinense* (MFLU 20-0433). **(a–d)** Colonies on MEA, CYA, PDA and rosebengal at 25°C, respectively. **(e–j)** Conidiophores and conidia. **(k)** Conidia. Scale bars: **(e,f)** = 20 μm, **(g–k)** = 10 μm.

**FIGURE 11:**
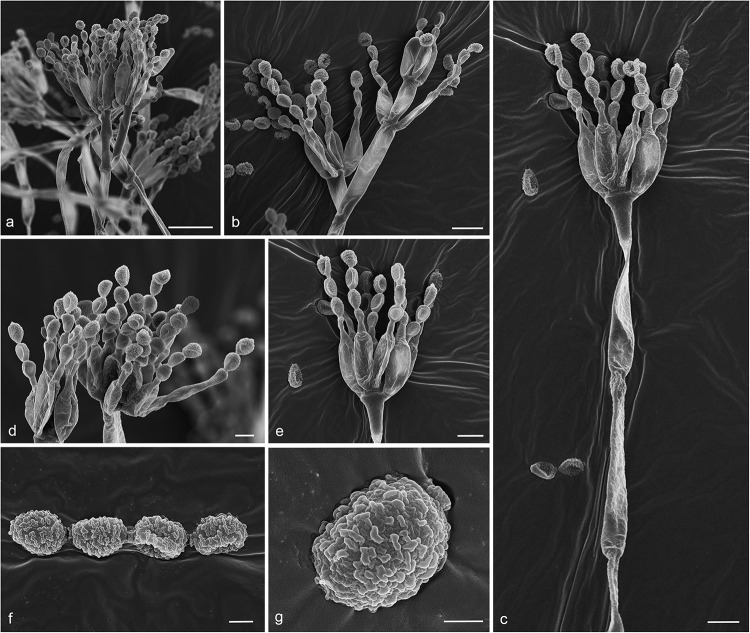
*Penicillium guaibinense* (MFLU 20-0433). **(a–c)** Conidiophores and conidia. **(d,e)** Catenate conidia forming on phialides. **(f)** Conidia. **(g)** Close-up of conidia showing conidial wall. Scale bars: **(a)** = 10 μm, **(b,c)** = 5 μm, **(d)** = 2 μm, **(e)** = 3 μm, **(f,g)** = 1 μm.

*Facesoffungi number*: FoF 08839

*Macromorphology*: *Colonies on MEA* reaching 55–60 mm diam. after 7 days in the dark at 25°C, circular to slightly undulate, with entire margin, flat or effuse, dense, with wrinkled from center to edge, white with pale yellow circle from above, pale yellow from below. *Colonies on CYA* reaching 30–35 mm diam. after 7 days in the dark at 25°C, circular, filamentous, low convex in the center, flat at the edge, medium dense, cottony, white from above, orange-cream from below. *Colonies on PDA* reaching 25–32 mm diam. after 7 days in the dark at 25°C, circular, entire, flat or effuse, dense, cottony, white from above, yellow to cream from below. *Colonies on Rosebengal* reaching 40–45 mm diam. after 7 days in the dark at 25°C, circular, edge entire to filamentous, flat, dense, cottony, white from above and below.

*Micromorphology*: *Conidiophores* on MEA up to 200 μm long, 2.7–3.5 μm wide, divaricate, biverticillate to terverticillate, sometime quaterverticillate, stipes smooth- to finely verruculose-walled, septate, with branched 15–38 μm long × 2.6–3 μm wide. *Metulae* 11–20.3 × 2.2–4.1 μm ($\bar{x}$ = 13.7 × 3 μm, *n* = 20), 1–3 per branch, divergent, each bearing 3–10 phialides. *Phialides* 6.8–14.6 × 2.5–3.4 μm ($\bar{x}$ = 9.8 × 2.8 μm, *n* = 20), ampulliform to cylindrical. *Conidia* 2.8–4 × 2.5–3.9 μm ($\bar{x}$ = 3.6 × 3.2 μm, *n* = 30), hyaline, globose, subglobose to ellipsoidal, catenate, verruculose, finely rough-walled.

*Material examined*: CHINA, Yunnan Province, Honghe County, from rhizosphere soil of *Quercus rubra*, 16 April 2018, M. Doilom (MFLU 20-0433); living culture (KUMCC 18-0199).

**FIGURE 12:**
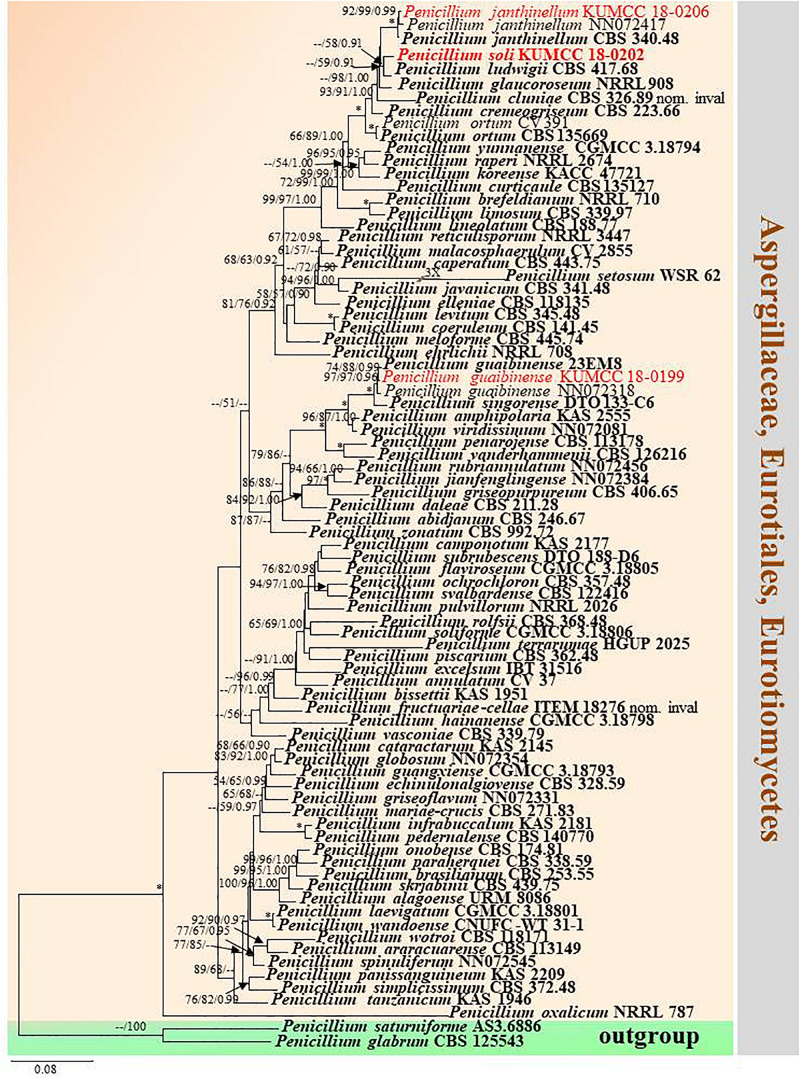
Phylogram generated from maximum likelihood analysis based on combined ITS, *BenA*, *CaM*, and *RPB2* sequence data. Eighty-one sequences are included in the combined analysis, which consisted of 2590 characters including alignment gaps. The tree is rooted to *Penicillium glabrum* (CBS 125543) and *P. saturniforme* (AS3.6886). The MP analysis for the combined dataset had 815 parsimony informative, 1358 constant, 417 parsimony uninformative characters and yielded 39 most parsimonious trees. The best-fit model GTR + I + G was selected for ITS, *BenA* and *RPB2*, and SYM + I + G for *CaM* in BI analysis. Maximum parsimony and maximum likelihood bootstrap values ≥ 50% and Bayesian posterior probabilities ≥ 0.90 (MPBS/MLBS/BYPP) are indicated at the nodes. Branches with 100% MPBS, 100% MLBS and 1.00 BYPP values are indicated as an asterisk (^∗^). The ex-type strains are bolded black, and the new isolates are in red.

*Notes*: Conidia of our specimen MFLU 20-0433 are slightly wider and longer than the holotype of *Penicillium guaibinense* [2.8–4 × 2.5–3.9 μm (av. 3.6 × 3.2) vs. 2–3 × 2–3 μm (av. 2.3 × 2.2)]. However, it is identified as *P. guaibinense* based on phylogenetic analysis of combined ITS, *BenA*, *CaM*, and *RPB2* sequences (Figure 12) and nucleotide comparison of ITS, *BenA* and *CaM* (Supplementary Table S4).

Crous et al. (2018) established *Penicillium guaibinense* from soil in the Guaibim sandbank in Bahia, Brazil. Diao et al. (2018) introduced *P. austrosinense* from acidic soil in Hainan, China. The conidia of *P. guaibinense* (CCDCA 11512 = 23EM8: ex-type) are finely rough and broadly subglobose, whereas they are smooth-walled and globose to ellipsoidal in *P. austrosinense* (CGMCC 3.18797: ex-type); despite this, conidial sizes between *P. guaibinense* and *P. austrosinense* are slightly similar [2–3 × 2–3 μm (av. 2.3 × 2.2) vs. 2–4 × 2–3 μm (av. 2.87 × 2.59)]. *Penicillium austrosinense* is considered as a synonym of *P. guaibinense* on the basis of phylogenetic analyses and nucleotide comparison of ITS, *BenA*, *CaM*, and *RPB2* (Supplementary Table S4). *Penicillium guaibinense* was prior published, and we treat *P. austrosinense* as a synonym of *P. guaibinense*.

#### *Penicillium soli* Doilom, C. F. Liao and D. Pem, sp. nov., Figure 13

*Index Fungorum number*: IF 557862; *Facesoffungi number*: FoF 08840

**FIGURE 13:**
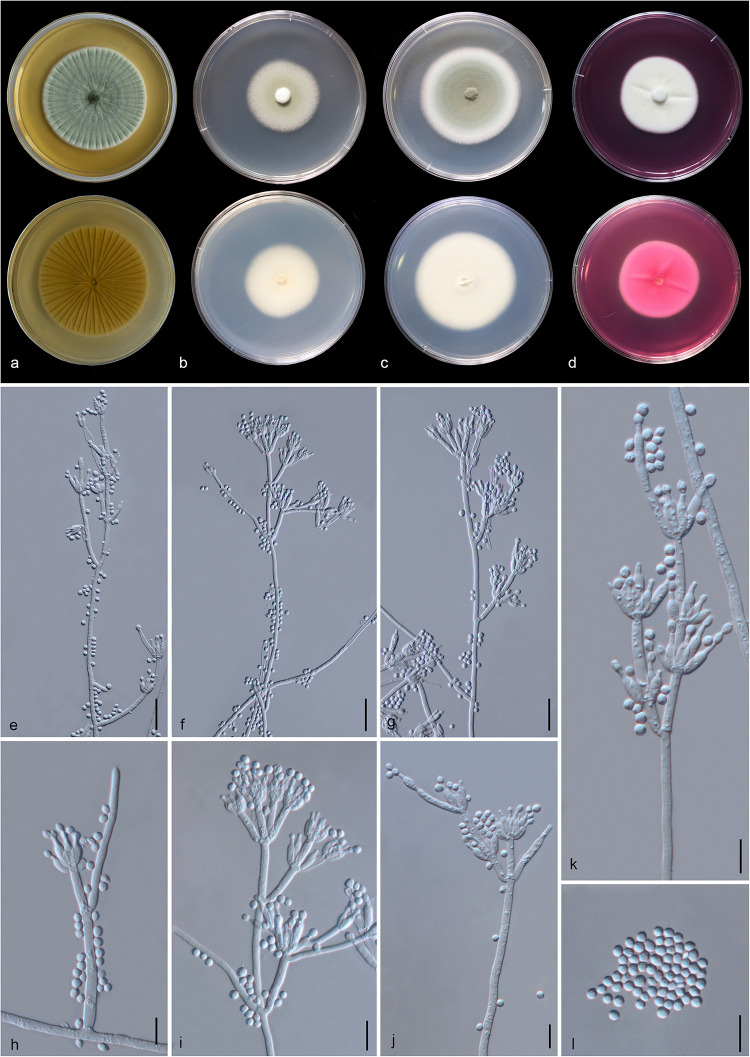
*Penicillium soli* (MFLU 20-0432, holotype). **(a–d)** Colonies on MEA, CYA, PDA and rosebengal at 25°C, respectively. **(e–g)** Conidiophores and conidia. **(h–k)** Close-up of conidiophores and conidia. **(l)** Conidia. Scale bars: **(e–g)** = 20 μm, **(h–l)** = 10 μm.

*Etymology*: The specific epithet “*soli*” From Latin, refers to soil, where the holotype was collected.

*Holotype*: MFLU 20-0432

*Macromorphology*: *Colonies on MEA* reaching 55–60 mm diam. after 7 days in the dark at 25°C, circular, edge entire, flat or effuse, dense, with wrinkled from center to edge, green with pale green to white at the edge from above, yellow or creamy with white at the edge from below. *Colonies on CYA* reaching 35–40 mm diam. after 7 days in the dark at 25°C, circular, edge entire, flat, medium dense, gray in the center, white at the edge from above and white from below. *Colonies on PDA* reaching 53–55 mm diam. after 7 days in the dark at 25°C, circular, edge entire, flat or effuse, dense, grayish in the center gradually green and white at the edge from above and white from below. *Colonies on Rosebengal* reaching 40–45 mm diam. after 7 days in the dark at 25°C, circular, edge entire, flat, dense, cottony, with slightly wrinkled from center to edge, white from above and below.

*Micromorphology*: *Sclerotia* absent. *Conidiophores* on MEA up to 205 μm long, 2.7–4.2 μm wide, divaricate, biverticillate, sometime terverticillate to quaterverticillate, stipes smooth-walled, septate, with branches 17–51 × 3–4.7 μm. *Metulae* 11–23 × 2.3–4.5 μm ($\bar{x}$ = 15 × 3.1 μm, *n* = 30), divergent, 1–4 per branch, each bearing 3–9 phialides, with 2–3 whorls per branch. *Phialides* 6.5–12.3 × 2.2–3.5 μm ($\bar{x}$ = 8.7 × 2.8 μm, *n* = 30), ampulliform to cylindrical. *Conidia* 2.5–4.5 × 2.3–3.4 μm ($\bar{x}$ = 3.4 × 2.8 μm, *n* = 30), hyaline, globose, subglobose to broadly ellipsoidal, chain, smooth-walled.

*Material examined*: CHINA, Yunnan Province, Honghe County, from rhizosphere soil of *Quercus rubra*, 16 April 2018, M. Doilom (MFLU 20-0432, **holotype**); ex-type living culture (KUMCC 18-0202).

*Notes*: In the phylogram generated from maximum likelihood analysis based on ITS sequence data, *P. soli* was closely related to *Penicillium* spp., e.g., *P. cremeogriseum* (CBS 223.66) (Supplementary Figure S5). The new taxon *P. soli* clustered with *P. ludwigii* (CBS 417.68) in the analysis based on *BenA* sequences (Supplementary Figure S6). Upon analysis of the *CAM* and *RPB2* sequence data, *P. soli* clustered as a sister to *P. cluniae* (CBS 326.89) (Supplementary Figures S7, S8), and showed 0.96% (4/415) and 1.46% (11/755) nucleotide differences, respectively. However, phylogenetic analyses across a combined ITS, *BenA*, *CAM* and *RPB2* sequence data showed that *P. soli* (KUMCC 18-0202) formed a distinct lineage and was sister to *P. ludwigii* (CBS 417.68), with bootstrap support in ML analysis (58% ML, 0.91 PP) (Figure 12). Comparison of *CaM* and *RPB2* sequence data between *P. soli* (KUMCC 18-0202) and *P. ludwigii* (CBS 417.68) indicated 5.3% (22/415) and 2.38% (18/755) nucleotide difference, respectively. Accordingly, following the guidelines of Jeewon and Hyde (2016), we introduce *P. soli* as a new species.

### *Talaromyces* Section *Talaromyces* C. R. Benj.

= *Penicillium* subgenus *Biverticillium* section *Simplicium* series *Mini- olutea* Pitt, The genus *Penicillium*: 419. 1980.

= *Penicillium* subgenus *Biverticillium* section *Coremigenum* series

*Duclauxii* Raper and Thom ex Pitt, The genus *Penicillium*: 404. 1980.

= *Talaromyces* section *Talaromyces* series *Flavi* Pitt, The genus *Penicillium*: 471. 1980.

*Type*: *Talaromyces flavus* (Klöcker) Stolk and Samson

*Notes*: *Talaromyces* section *Talaromyces* was introduced by Stolk and Samson (1972) and characterized by yellow ascomata, as well as occasionally white, creamish, pinkish or reddish and yellow ascospores. Conidiophores are usually of the biverticillate-symmetrical type, and some species are reduced to conidiophores with solitary phialides. Phialides are usually acerose, with a minor proportion of species having wider bases (Stolk and Samson, 1972). Phylogenetic analyses of ITS, *BenA*, *CaM*, and *RPB2* sequence data were used to determine species delimitation (Barbosa et al., 2018; Guevara-Suarez et al., 2020). Section *Talaromyces* species are usually isolated from soil, indoor air environments and humans (Chen et al., 2016; Yilmaz et al., 2016; Lau et al., 2017; Pangging et al., 2019; Sun et al., 2020). We introduce the new species *T. yunnanensis* into the section.

#### *Talaromyces yunnanensis* Doilom and C. F. Liao, sp. nov., Figure 14

*Index Fungorum number*: IF 557863; *Facesoffungi number*: FoF 08841

**FIGURE 14:**
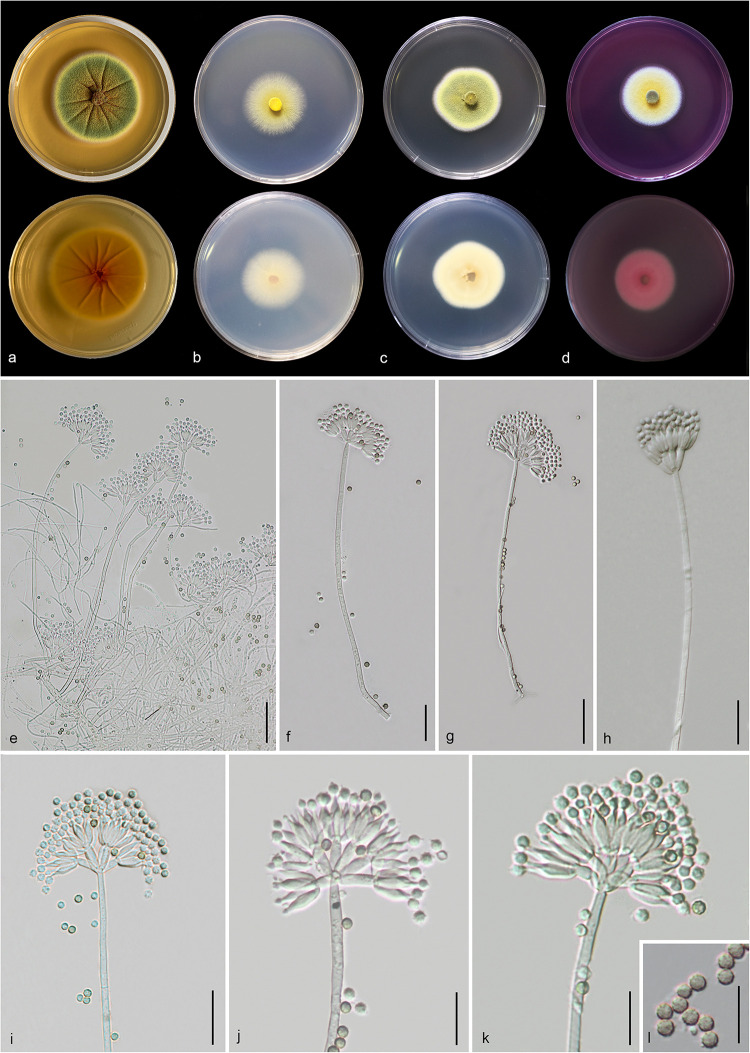
*Talaromyces yunnanensis* (MFLU 20-0434, holotype). **(a–d)** Colonies on MEA, CYA, PDA and rosebengal at 25°C, respectively. **(e–h)** Conidiophores and conidia. **(i–k)** Close-up of conidiophores and conidia. **(l)** Conidia. Scale bars: **(e,g)** = 30 μm, **(f,h,i)** = 20 μm, **(j–l)** = 5 μm.

*Etymology*: Referring to the province, where the holotype was collected.

*Holotype*: MFLU 20-0434

*Macromorphology*: *Colonies on MEA* reaching 50–53 mm diam. after 7 days in the dark at 25°C, circular, edge entire, flat or effuse, dense, wrinkled from center to edge, green with orange mass (exudate) in the center, gradually yellow and white at the edge from above, yellow from below. *Colonies on CYA* reaching 35–37 mm diam. after 7 days in the dark at 25°C, circular, edge entire to filamentous, flat, medium dense, yellow at the center and white at the edge from above, pale yellow at the center and white at the edge from below. *Colonies on PDA* reaching 30–40 mm diam. after 7 days in the dark at 25°C, irregular in shape, curled, flat, dense, yellowish green in the center, yellow and white from above, yellowish cream from below. *Colonies on Rosebengal* reaching 35–38 mm diam. after 7 days in the dark at 25°C, circular, edge entire, flat, dense, yellow in the center, white at the edge from above, white from below.

*Micromorphology*: *Sclerotia* absent. *Conidiophores* on MEA up to 275 μm long, 2.2–3.7 μm wide, biverticillate, stipes finely verruculose-walled, septate, mostly unbranched, occasionally with branched 9.5–10 μm long × 2.5–3.1 μm wide. *Metulae* 7.3–11.5 × 2.3–4.2 μm ($\bar{x}$ = 9.3 × 3.2 μm, *n* = 20), 4–8 per stipe, divergent, each bearing 3–10 phialides. *Phialides* 7.3–11.5 × 2.2–3.8 μm ($\bar{x}$ = 9.4 × 2.9 μm, *n* = 30), ampulliform to cylindrical. *Conidia* 2.3–3.4 × 2.1–3.2 μm ($\bar{x}$ = 2.8 × 2.6 μm, *n* = 30), hyaline when immature, pale brown to dark brown when mature, globose to subglobose, chain, finely verruculose-walled. *Ascomata* not observed.

*Material examined*: CHINA, Yunnan Province, Honghe County, from rhizosphere soil of *Quercus rubra*, 16 April 2018, M. Doilom (MFLU 20-0434, **holotype**); ex-type living culture (KUMCC 18-0208).

**FIGURE 15:**
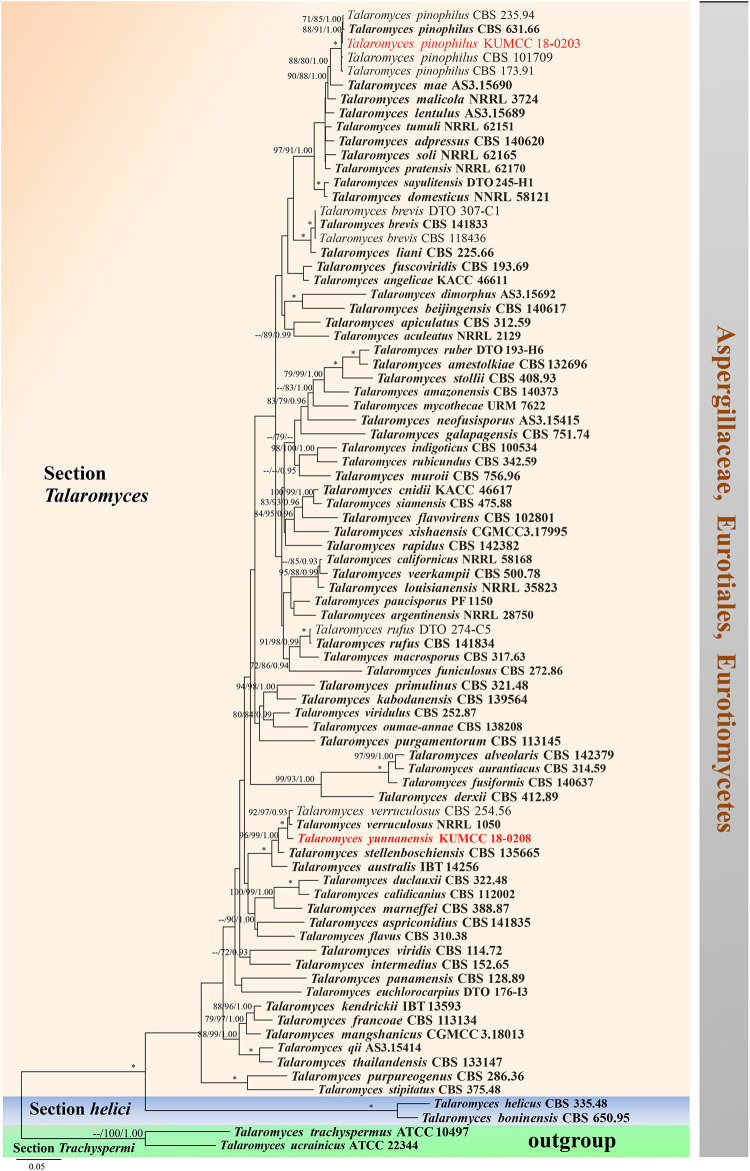
Phylogram generated from maximum likelihood analysis based on combined ITS, *BenA*, *CaM*, and *RPB2* sequence data. Eighty-two sequences are included in the combined analysis, which consisted of 2858 characters including alignment gaps. The tree is rooted to *Talaromyces trachyspermus* (ATCC 10497) and *T. ucrainicus* (ATCC 22344). The MP analysis for the combined dataset had 857 parsimony informative, 1552 constant, 176 parsimony uninformative characters and yielded 46 most parsimonious trees. The best-fit model SYM + I + G was selected for *CaM*, GTR + I + G for ITS and HKY + I + G for *RPB2* and *BenA* in BI analysis. Maximum parsimony and maximum likelihood bootstrap values ≥ 70% and Bayesian posterior probabilities ≥ 0.90 (MPBS/MLBS/BYPP) are indicated at the nodes. Branches with 100% MPBS, 100% MLBS and 1.00 BYPP values are indicated as an asterisk (^∗^). The ex-type strains are bolded black, and the new isolates are in red.

*Notes*: *Talaromyces yunnanensis*, *T. verruculosus*, and *T. stellenboschiensis* have similar morphological characteristics featuring biverticillate conidiophores, ampulliform phialides tapering into thin necks and globose conidia; however, they can be differentiated by colony characteristics. *T. yunnanensis* grows faster than *T. stellenboschiensis* (50–53 vs. 40–42 mm) and *T. verruculosus* (50–53 vs. 35–36 mm) after 7 days of incubation on MEA at 25°C (Visagie et al., 2015). *T. yunnanensis* (KUMCC 18-0208) was phylogenetically related to *T. verruculosus* (NRRL 1050: ex-type) and (CBS 254.56) but had its own distinct linear with 100% MP, 100% ML, 1.00 PP (Figure 15). Phylogenetic analysis based on individual ITS sequence data was unsuccessful in separating *T. yunnanensis* and *T. verruculosus* (Supplementary Figure S9). However, *BenA* and *CaM* were able to separate these two species (Supplementary Figures S10, S11). The *RBP2* gene was amplified and sequenced but also unsuccessful. Thus, comparison was unable to be performed. Comparison of *BenA* and *CaM* sequence data between *T. yunnanensis* (KUMCC 18-0208) and *T. verruculosus* (NRLL 1050: ex-type) revealed 1.94% (9/465) and 2.08% (10/480) nucleotide difference, respectively. This justifies these two isolates as different taxa according to the guidelines proposed by Jeewon and Hyde (2016).

## Discussion

We carried out phosphate solubilization of 13 fungal strains in both solid and liquid PVK media *in vitro*. This study indicates that the airborne isolate KUMCC 18-0196 (*A. hydei* sp. nov.) was a highly efficient strain in solubilizing phosphate. Isolate KUMCC 18-0196 also showed the greatest drop in pH in the PVK broth containing TCP, suggesting that it might produce organic acids. The *A. hydei* is the most efficient P-solubilizing than other strains tested in this study. Our results are similar to the study of Pradhan and Sukla (2006), which concluded that *Aspergillus* spp. show a much higher drop in pH in the PVK broth containing TCP and high P solubilization when compared to *Penicillium* spp. Many previous studies reported PSF mostly from soils, including rhizospheric and non-rhizospheric soil samples from cultivated plants and asymptomatic plant roots (Nelofer et al., 2015; Elias et al., 2016; Adhikari and Pandey, 2019; Qiao et al., 2019, Supplementary Table S5). To date, PSF from air have not yet been reported. Thus, *A. hydei* sp. nov. described herein is the first report of PSF from air. However, the determination of phosphate solubilization has been tested on solid and liquid media under laboratory conditions. The ability to solubilize phosphate under field conditions should be closely examined to confirm P dissolving activity. PSF did not lose P solubilizing ability under laboratory conditions, although sub-culturing was repeated (Kucey, 1983; Sharma et al., 2013).

Several phosphate-solubilizing microorganisms viz. actinomyces, bacteria, fungi, and yeasts have been screened for phosphate solubilization. They were shown to enhance the solubilization of insoluble P compounds (Gizaw et al., 2017; Nandimath et al., 2017; Mohamad et al., 2018; Wang et al., 2018; Chen and Liu, 2019; Liu et al., 2019). For fungi, *Rhizopus stolonifer* var. *stolonifer*, *Aspergillus niger*, and *Alternaria alternata* have been found in the literature to be the most effective strains with respect to the amount of TCP solubilization by Ceci et al. (2018). However, solubilization by PSF depends on the insoluble inorganic phosphate source, type of carbon, nitrogen and metal ions in soils and culture conditions (Richardson and Simpson, 2011; Jyoti et al., 2013). Vyas et al. (2007) tested the quantity of phosphate solubilized by *Eupenicillium parvum* (current name *Penicillium parvum*) from the different inorganic phosphates [TCP, aluminum phosphate (AP) and North Carolina phosphate (NCRP)]. Their results showed that the quantity of phosphate solubilized was by far the greatest for TCP among the inorganic phosphates, whereas the solubilization of iron phosphate was low. Phosphate-solubilizing microorganisms convert insoluble phosphates into a soluble form through the processes of acidification, chelation, exchange reaction, and production of organic acid (Behera et al., 2017).

Some studies of PSF identified fungal species names based on morphology, highest nucleotide similarity in MegaBLAST searches of NCBI’s GenBank nucleotide database and/or phylogenetic analysis of ITS sequence data (Nelofer et al., 2015; Wang et al., 2018; Zhang et al., 2018, Supplementary Table S5). However, these are insufficient to determine the species names, especially within species complexes (Samson et al., 2014; Doilom et al., 2017; Hyde et al., 2020a,b). A comparison of sequence data from fungal strains with the ex-type cultures of named species must be considered to confirm the species name (Dayarathne et al., 2016). Several taxonomic schemes and new molecular approaches have been proposed for fungal identification (Visagie et al., 2014b; Jeewon and Hyde, 2016; Zhao et al., 2018). We provide the fungal identification of PSF based on morphological characteristics and multi-loci phylogenetic analyses, including the GCPSR method where necessary. GenBank accession numbers of species in *Aspergillus*, *Gongronella*, *Penicillium*, and *Talaromyces* with our newly generated sequences are also provided in Supplementary Table S1 to further facilitate species identification. Fungal taxonomy is discussed in each taxonomic note.

Applications of beneficial microbes, especially phosphate-solubilizing fungi, are useful toward plant growth-promoting strategies. The isolate KUMCC 18-0196 (*A. hydei* sp. nov.) was a highly efficient strain in solubilizing phosphate; accordingly, we are optimistic that it can play potential roles in supporting plant growth in practical applications. This research provides important information relevant to the application of beneficial fungi to promote plant growth. This will have critical implications for the development of agriculture, industry, restoration ecology and forestry going forward.

## Data Availability Statement

The datasets presented in this study can be found in online repositories. The names of the repository/repositories and accession number(s) can be found in the article/Supplementary Material.

## Author Contributions

MD, PM, J-CX, and J-WG designed the study. MD planned and conducted the experiments and wrote the manuscript. MD, KY, WD, C-FL, RP, KY, and DP conducted the experiments, analyzed the data, and revised the manuscript. PM, IP, NS, and SL contributed to research funds. All authors revised the manuscript.

## Conflict of Interest

The authors declare that the research was conducted in the absence of any commercial or financial relationships that could be construed as a potential conflict of interest.

## Footnotes

1 http://blast.ncbi.nlm.nih.gov/

2 http://mafft.cbrc.jp/alignment/software/

3 www.splitstree.org

## Supplementary Material

Supplementary Figure S1 Phylogram generated from maximum likelihood analysis based on ITS sequence data of *Aspergillus* strains.

Supplementary Figure S2 Phylogram generated from maximum likelihood analysis based on *CaM* sequence data of *Aspergillus* strains.

Supplementary Figure S3 Phylogram generated from maximum likelihood analysis based on *BenA* sequence data of *Aspergillus* strains.

Supplementary Figure S4 Phylogram generated from maximum likelihood analysis based on *RPB2* sequence data of *Aspergillus* strains.

Supplementary Figure S5 Phylogram generated from maximum likelihood analysis based on ITS sequence data of *Penicillium* strains.

Supplementary Figure S6 Phylogram generated from maximum likelihood analysis based on *BenA* sequence data of *Penicillium* strains.

Supplementary Figure S7 Phylogram generated from maximum likelihood analysis based on *CaM* sequence data of *Penicillium* strains.

Supplementary Figure S8 Phylogram generated from maximum likelihood analysis based on *RPB*2 sequence data of *Penicillium* strains.

Supplementary Figure S9 Phylogram generated from maximum likelihood analysis based on ITS sequence data of *Talaromyces* strains.

Supplementary Figure S10 Phylogram generated from maximum likelihood analysis based on *BenA* sequence data of *Talaromyces* strains.

Supplementary Figure S11 Phylogram generated from maximum likelihood analysis based on *CaM* sequence data of *Talaromyces* strains.

Supplementary Table S1 Taxa and their GenBank accession numbers used in this study.

Supplementary Table S2 Phosphate solubilization of tricalcium phosphate (TCP) on solid and liquid Pikovskaya (PVK) media with pH values by 13 fungal strains and control.

Supplementary Table S3 Details of 13 phosphate-solubilizing fungal strains used in this study.

Supplementary Table S4 Nucleotide comparison of ITS, *BenA*, *CaM*, and *RPB2* between strains of *Penicillium guaibinense* and *P. austrosinense*.

Supplementary Table S5 List of phosphate-solubilizing fungi.
