# Supplementary material for: Screening of Phosphate-Solubilizing Fungi From Air and Soil in Yunnan, China: Four Novel Species in Aspergillus, Gongronella, Penicillium, and Talaromyces
Source: Front Microbiol. 2020 Oct 6;11:585215. doi: 10.3389/fmicb.2020.585215 (PMC7574596; doi:10.3389/fmicb.2020.585215)
Supplement: Supplementary Figure S1 — Phylogram generated from maximum likelihood analysis based on ITS sequence data of Aspergillus strains. [file Data_Sheet_2.doc]

**FIGURE S1** Phylogram generated from maximum likelihood analysis based on ITS sequence data of *Aspergillus* strains. Eighty-six sequences are included in the ITS analysis, which consisted of 556 characters including alignment gaps. The RAxML analysis resulted in a best scoring likelihood tree selected with a final combined dataset = -3231.969399. The matrix had 251 distinct alignment patterns, with 9.84 % of undetermined characters or gaps. Estimated base frequencies were as follows; A = 0.181276, C = 0.314668, G = 0.285346, T = 0.218710; substitution rates AC = 2.173999, AG = 2.556724, AT = 2.547258, CG = 0.761946, CT = 5.477120, GT = 1.000000; gamma distribution shape parameter α = 0.262997; tree length = 1.487623. The tree is rooted to *Penicillium viridissimum* (CGMCC 3.18796) and *P. laevigatum* (CGMCC 3.18801). Maximum likelihood bootstrap values ≥ 70 % are indicated at the nodes. The ex-type strains are bolded black, and the new isolates are highlighted with colored blocks.

**FIGURE S2** Phylogram generated from maximum likelihood analysis based on *CaM* sequence data of *Aspergillus* strains. One hundred and one sequences are included in the *CaM* analysis, which consisted of 566 characters including alignment gaps. The RAxML analysis resulted in a best scoring likelihood tree selected with a final combined dataset = -9300.997146. The matrix had 455 distinct alignment patterns, with 20.67 % of undetermined characters or gaps. Estimated base frequencies were as follows; A = 0.257348, C = 0.245572, G = 0.247213, T = 0.249866; substitution rates AC = 1.062744, AG = 4.043370, AT = 1.167052, CG = 1.002847, CT = 6.079218, GT = 1.000000; gamma distribution shape parameter α = 0.579314; tree length = 5.317884. The tree is rooted to *Penicillium viridissimum* (CGMCC 3.18796) and *P. laevigatum* (CGMCC 3.18801). Maximum likelihood bootstrap values ≥ 70 % are indicated at the nodes. The ex-type strains are bolded black, and the new isolates are highlighted with colored blocks.

**FIGURE S3** Phylogram generated from maximum likelihood analysis based on *BenA* sequence data of *Aspergillus* strains. One hundred sequences are included in the *BenA* analysis, which consisted of 670 characters including alignment gaps. The RAxML analysis resulted in a best scoring likelihood tree selected with a final combined dataset = -8608.444765. The matrix had 435 distinct alignment patterns, with 17.34 % of undetermined characters or gaps. Estimated base frequencies were as follows; A = 0.214299, C = 0.282885, G = 0.257493, T = 0.245323; substitution rates AC = 0.941981, AG = 3.241155, AT = 1.076131, CG = 0.854959, CT = 3.517670, GT = 1.000000; gamma distribution shape parameter α = 0.529998; tree length = 5.470814. The tree is rooted to *Penicillium viridissimum* (CGMCC 3.18796) and *P. laevigatum* (CGMCC 3.18801). Maximum likelihood bootstrap values ≥ 70 % are indicated at the nodes. The ex-type strains are bolded black, and the new isolates are highlighted with colored blocks.

**FIGURE S4** Phylogram generated from maximum likelihood analysis based on *RPB2* sequence data of *Aspergillus* strains. Seventy-four sequences are included in the *RPB2* analysis, which consisted of 1013 characters including alignment gaps. The RAxML analysis resulted in a best scoring likelihood tree selected with a final combined dataset = -8534.449728. The matrix had 435 distinct alignment patterns, with 2.49 % of undetermined characters or gaps. Estimated base frequencies were as follows; A = 0.247523, C = 0.253854, G = 0.266300, T = 0.232324; substitution rates AC = 0.804492, AG = 4.753025, AT = 1.026365, CG = 1.116284, CT = 7.334808, GT = 1.000000; gamma distribution shape parameter α = 0.208190; tree length = 1.896136. The tree is rooted to *Penicillium viridissimum* (CGMCC 3.18796) and *P. laevigatum* (CGMCC 3.18801). Maximum likelihood bootstrap values ≥ 70 % are indicated at the nodes. The ex-type strains are bolded black, and the new isolates are highlighted with colored blocks.

**FIGURE S5** Phylogram generated from maximum likelihood analysis based on ITS sequence data of *Penicillium* strains. Eighty sequences are included in the ITS analysis, which consisted of 565 characters including alignment gaps. The RAxML analysis resulted in a best scoring likelihood tree selected with a final dataset = -2662.050093. The matrix had 170 distinct alignment patterns, with 5.52 % of undetermined characters or gaps. Estimated base frequencies were as follows; A = 0.183664, C = 0.320563, G = 0.289418, T = 0.206356; substitution rates AC = 3.083390, AG = 2.541631, AT = 4.046378, CG = 0.529771, CT = 10.222389, GT = 1.000000; gamma distribution shape parameter α = 0.167930; tree length = 1.395086. The tree is rooted to *Penicillium glabrum* (CBS 125543) and *P. saturniforme* (AS3.6886). Maximum likelihood bootstrap values ≥ 70 % are indicated at the nodes. The ex-type strains are bolded black, and the isolates in this study are highlighted with colored blocks.

**FIGURE S6** Phylogram generated from maximum likelihood analysis based on *BenA* sequence data of *Penicillium* strains. Eighty-one sequences are included in the *BenA* analysis, which consisted of 540 characters including alignment gaps. The RAxML analysis resulted in a best scoring likelihood tree selected with a final dataset = -6952.580939. The matrix had 350 distinct alignment patterns, with 17.74 % of undetermined characters or gaps. Estimated base frequencies were as follows; A = 0.220839, C = 0.299696, G = 0.236544, T = 0.242920; substitution rates AC = 1.216332, AG = 3.820563, AT = 1.725130, CG = 0.816566, CT = 3.895831, GT = 1.000000; gamma distribution shape parameter α = 0.425914; tree length = 3.855670. The tree is rooted to *Penicillium glabrum* (CBS 125543) and *P. saturniforme* (AS3.6886). Maximum likelihood bootstrap values ≥ 70 % are indicated at the nodes. The ex-type strains are bolded black, and the isolates in this study are highlighted with colored blocks.

**FIGURE S7** Phylogram generated from maximum likelihood analysis based on *CaM* sequence data of *Penicillium* strains. Seventy-eight sequences are included in the *CaM* analysis, which consisted of 557 characters including alignment gaps. The RAxML analysis resulted in a best scoring likelihood tree selected with a final dataset = -8450.631749. The matrix had 355 distinct alignment patterns, with 23.43 % of undetermined characters or gaps. Estimated base frequencies were as follows; A = 0.255306, C = 0.277203, G = 0.239825, T = 0.227666; substitution rates AC = 0.621412, AG = 2.500824, AT = 0.814136, CG = 0.577629, CT = 3.747050, GT = 1.000000; gamma distribution shape parameter α = 0.532795; tree length = 4.586437. The tree is rooted to *Penicillium glabrum* (CBS 125543) and *P. saturniforme* (AS3.6886). Maximum likelihood bootstrap values ≥ 70 % are indicated at the nodes. The ex-type strains are bolded black, and the isolates in this study are highlighted with colored blocks.

**FIGURE S8** Phylogram generated from maximum likelihood analysis based on *RPB2* sequence data of *Penicillium* strains. Seventy-eight sequences are included in the *RPB2* analysis, which consisted of 755 characters including alignment gaps (*RPB2* sequence data of *Penicillium* *setosum* is not included). The RAxML analysis resulted in a best scoring likelihood tree selected with a final dataset = -7223.216349. The matrix had 285 distinct alignment patterns, with 0.79 % of undetermined characters or gaps. Estimated base frequencies were as follows; A = 0.235195, C = 0.269864, G = 0.263543, T = 0.231398; substitution rates AC = 1.395748, AG = 5.956521, AT = 1.557643, CG = 1.133266, CT = 11.417774, GT = 1.000000; gamma distribution shape parameter α = 0.174880; tree length = 2.165217. The tree is rooted to *Penicillium glabrum* (CBS 125543) and *P. saturniforme* (AS3.6886). Maximum likelihood bootstrap values ≥ 70 % are indicated at the nodes. The ex-type strains are bolded black, and the isolates in this study are highlighted with colored blocks.

**FIGURE S9** Phylogram generated from maximum likelihood analysis based on ITS sequence data of *Talaromyces* strains. Eighty-two sequences are included in the ITS analysis, which consisted of 584 characters including alignment gaps. The RAxML analysis resulted in a best scoring likelihood tree selected with a final dataset = 2959.110959. The matrix had 205 distinct alignment patterns, with 10.51 % of undetermined characters or gaps. Estimated base frequencies were as follows; A = 0.189192, C = 0.298558, G = 0.294101, T = 0.218149; substitution rates AC = 1.838764, AG = 1.711981, AT = 1.308565, CG = 0.258757, CT = 9.028585, GT = 1.000000; gamma distribution shape parameter α = 0.167094; tree length = 2.437036. The tree is rooted to *Talaromyces trachyspermus* (ATCC 10497) and *T. ucrainicus* (ATCC 22344). Maximum likelihood bootstrap values ≥ 70 % are indicated at the nodes. The ex-type strains are bolded black, and the isolates in this study are highlighted with colored blocks.

**FIGURE S10** Phylogram generated from maximum likelihood analysis based on *BenA* sequence data of *Talaromyces* strains. Eighty-one sequences are included in the *BenA* analysis, which consisted of 482 characters including alignment gaps. The RAxML analysis resulted in a best scoring likelihood tree selected with a final dataset = -6321.914252. The matrix had 314 distinct alignment patterns, with 21.24 % of undetermined characters or gaps. Estimated base frequencies were as follows; A = 0.233926, C = 0.291522, G = 0.220463, T = 0.254090; substitution rates AC = 1.603824, AG = 4.772181, AT = 1.292647, CG = 1.211163, CT = 4.277297, GT = 1.000000; gamma distribution shape parameter α = 0.395921; tree length = 4.362224. The tree is rooted to *Talaromyces trachyspermus* (ATCC 10497) and *T. ucrainicus* (ATCC 22344). Maximum likelihood bootstrap values ≥ 70 % are indicated at the nodes. The ex-type strains are bolded black, and the isolates in this study are highlighted with colored blocks.

**FIGURE S11** Phylogram generated from maximum likelihood analysis based on *CaM* sequence data of *Talaromyces* strains. Seventy-nine sequences are included in the *CaM* analysis, which consisted of 613 characters including alignment gaps. The RAxML analysis resulted in a best scoring likelihood tree selected with a final dataset = -9289.463569. The matrix had 390 distinct alignment patterns, with 24.04 % of undetermined characters or gaps. Estimated base frequencies were as follows; A = 0.286991, C = 0.232256, G = 0.250088, T = 0.230664; substitution rates AC = 0.766118, AG = 2.388558, AT = 1.042737, CG = 0.625002, CT = 4.592605, GT = 1.000000; gamma distribution shape parameter α = 0.411179; tree length = 6.731057. The tree is rooted to *Talaromyces trachyspermus* (ATCC 10497) and *T. ucrainicus* (ATCC 22344). Maximum likelihood bootstrap values ≥ 70 % are indicated at the nodes. The ex-type strains are bolded black, and the isolates in this study are highlighted with colored blocks.
