# Supplementary material for: Screening of Phosphate-Solubilizing Fungi From Air and Soil in Yunnan, China: Four Novel Species in Aspergillus, Gongronella, Penicillium, and Talaromyces
Source: Front Microbiol. 2020 Oct 6;11:585215. doi: 10.3389/fmicb.2020.585215 (PMC7574596; doi:10.3389/fmicb.2020.585215)
Supplement: Supplementary Table S1 — Taxa and their GenBank accession numbers used in this study. [file Data_Sheet_1.doc]

**TABLE S1** Taxa and their GenBank accession numbers used in this study.

| **Section** | **Taxa** | **Strains^a^** | **GenBank accession numbers** | | | |
| --- | --- | --- | --- | --- | --- | --- |
|  |  |  | **ITS** | **BenA** | **CaM** | **RPB2** |
| *Flavi* | *Aspergillus aflatoxiformans* | DTO 228-G2 | MG662388 | MG517706 | MG518076 | MG517897 |
|  | *A. arachidicola* | ^T^ CBS 117610 | EF409241 | EF203158 | EF202049 | MG517802 |
|  | *A. austwickii* | ^HT^ DTO 228-F7 | MG662391 | MG517702 | MG518072 | MG517893 |
|  | *A. cerealis* | ^HT^ DTO 228-E7 | MG662394 | MG517693 | MG518063 | MG517884 |
|  | *A. flavus* | ^T^ NRRL 1957 (ex-type) | AF027863 | EF661485 | EF661508 | EF661440 |
|  | *A. flavus* | ^NT^ CBS 501.65 (ex neotype of *A. subolivaceus*) | EF661563 | MG517642 | MG518015 | MG517827 |
|  | *A. flavus* | ^NT^ NRRL 447 (ex-neotype of *A. oryzae*) | EF661560 | EF661483 | EF661506 | EF661438 |
|  | ***A. flavus*** | **KUMCC 18-0200** | **MT152329** | **MT161676** | **MT178244** | **MT384367** |
|  | ***A. flavus*** | **KUMCC 18-0207** | **MT152330** | **MT161677** | **MT178245** | **MT384368** |
|  | *A. minisclerotigenes* | ^T^ CBS 117635 | EF409239 | KY924667 | MG518009 | MG517799 |
|  | *A. mottae* | ^HT^ CBS 130016 | JF412767 | MG517687 | MG518058 | MG517878 |
|  | *A. parasiticus* | ^HT^ CBS 100926 = IBT 32309 = MUM 10.231 | AF027862 | EF661481 | EF661516 | EF661449 |
|  | *A. pipericola* | ^HT^ CBS 143680 = DTO 228-H4 = IBT 24628 | MG662385 | MG517717 | MG518087 | MG517908 |
|  | *A. pseudotamarii* | ^HT^ CBS 766.97 = NRRL 25517 = IBT 21092 | AF272574 | EF203125 | EF202030 | EU021631 |
|  | *A. sergii* | ^HT^ CBS 130017 = MUM 10.219 = IBT 32292 = IBT 32293 | KY937936 | MG517688 | MG518059 | MG517879 |
|  | *A. sojae* | ^NT^ CBS 100928 = DTO 046-C3 = IMI 191300 | KJ175434 | KJ175494 | KJ175550 | MG517831 |
|  | *A. subflavus* | ^HT^ CBS 143683 = DTO 326-E8 = S778 = CCF 4957 = NRRL 66254 = IBT 34939 | MH279429 | MG517773 | MG518143 | MG517964 |
|  | *A. tamarii* | ^LT^ NRRL 20818 | AF004929 | EF661474 | EF661526 | EU021629 |
| *Fumigati* | *A. arcoverdensis* | ^HT^ IFM 61334 = JCM 19878 | N/A | AB818845 | AB818856 | LC367679 |
|  | *A. assulatus* | ^HT^ KACC 41691 = IBT 27911 | HF545007 | DQ114123 | DQ114131 | HF545311 |
|  | *A. aureolus* | ^LT^ NRRL 2244 | EF669950 | EF669808 | EF669877 | EF669738 |
|  | *A. auratus* | ^T^ CBS 466.65 = NRRL 4378 = ATCC 16894 = IFO 8783 = IMI 075886 = QM 7860 = WB 4378 | EF669979 | EF669835 | EF669905 | EF669766 |
|  | *A. australensis* | ^HT^ CBS 112.55 = NRRL 2392 = IMI 061450 = WB 2392 | EF669953 | EF669811 | EF669880 | EF669741 |
|  | *A. brevipes* | ^IT^ NRRL 2439 | EF669954 | EF669812 | EF669881 | EF669742 |
|  | *A. denticulatus* | ^HT^ CBS 652.73 = KACC 41183 | N/A | DQ114125 | DQ114133 | LC367683 |
|  | *A. duricaulis* | ^NT^ CBS 481.65 = NRRL 4021 = ATCC 16900 = IMI 172282 = WB 4021 | EF669971 | EF669827 | EF669897 | EF669758 |
|  | *A. felis* | ^HT^ CBS 130245 = 131F4 | KF558318 | JX021700 | JX021715 | N/A |
|  | *A. fennelliae* | ^T^ CBS 598.74 = NRRL 5534 = ATCC 24325 = IMI 278382 = PIL605 | EF669994 | AF057320 | EF669920 | EF669781 |
|  | *A. ferenczii* | ^HT^ CBS 121594 = NRRL 4179 = IBT 27813 = DTO24-F2 | EF669977 | EF669833 | EF669903 | EF669764 |
|  | *A. ﬁscheri* | ^NT^ CBS 544.65 = NRRL 181 = ATCC 1020 = DSM 3700 = IMI 211391 = QM 1983 = Thom 4651.2 = WB 181 | EF669936 | EF669796 | EF669865 | EF669724 |
|  | *A. fumigatiaffinis* | ^HT^ IBT 12703 = CBS 117186 = KACC 41148 | N/A | DQ094885 | DQ094891 | N/A |
|  | *A. fumigatus* | ^T^ CBS 133.61 = NRRL 163 = ATCC 1022 = IMI 016152 = LSHBAc71 = NCTC 982 = QM 1981 = Thom 118 = WB 163 | EF669931 | EF669791 | EF669860 | EF669719 |
|  | ***A. fumigatus*** | **KUMCC 18-0197** | **MT152331** | **MT161678** | **MT178246** | **MT384369** |
|  | *A. galapagensis* | ^T^ CBS 117522 = IBT 16756 = KACC 41935 = DTO3H4 | N/A | DQ534145 | DQ534151 | LC367686 |
|  | *A. hiratsukae* | ^HT^ CBS 294.93 | MH862403 | N/A | AY870699 | N/A |
|  | *A. fumisynnematus* | ^HT^ IFM 42277 | AB250779 | AB248076 | AB259968 | LC367685 |
|  | *A. laciniosus* | ^T^ CBS 117721 = NRRL 35589 = KACC 41657 | AB299413 | AY870756 | AY870716 | N/A |
|  | *A. multiplicatus* | ^HT^ CBS 646.95 = IBT 17517 | N/A | DQ114129 | DQ114137 | N/A |
|  | *A. lentulus* | ^T^ CBS 117885 = NRRL 35552 = IBT 27201 = KACC 41940 | EF669969 | EF669825 | EF669895 | EF669756 |
|  | *A. nishimurae* | ^R^ CBS 117265 = IBT 3016 | HE974451 | DQ534154 | DQ534163 | HE974395 |
|  | *A. neoglaber* | ^NT^ CBS 111.55 = NRRL 2163 = ATCC 16909 = IFO 8789 = IMI 061447 = IMI 061447ii = QM 1903 = WB 2163 | EF669948 | EU014107 | EU014120 | EF669736 |
|  | *A. novofumigatus* | ^T^ CBS 117520 = KACC 41934 = IBT 16806 | N/A | DQ094886 | DQ094893 | N/A |
|  | *A. papuensis* | ^HT^ CBS 841.96 = IBT 27801 | EU220280 | AY870738 | AY870697 | LC367692 |
|  | *A. parafelis* | ^HT^ CM-3147 | N/A | KJ914692 | KJ914702 | KJ914731 |
|  | *N. paulistensis* | ^HT^ CBM-FA-0690 | N/A | AB488758 | AB488766 | N/A |
|  | *A. pseudofelis* | ^HT^ CM-6087 | N/A | KJ914697 | KJ914705 | KJ914734 |
|  | *A. pseudoviridinutans* | ^HT^ NIHAV1 | N/A | KJ914690 | KJ914708 | KJ914730 |
|  | *A. viridinutans* | ^ET^ CBS 127.56 = ATCC 16901 = IMI 062875 = IMI 062875ii = NRRL 4365 = WB 4081 = WB 4365 = WB 4782 | EF669978 | AF134779 | DQ534162 | EF669765 |
|  | *A. thermomutatus* | ^T^ NRRL 20748 = CBS 208.92 | EF669946 | EF669805 | EF669874 | EF669734 |
|  | *A. quadricinctus* | ^T^ CBS 135.52 = NRRL 2154 = ATCC 16897 = IMI 48583 = IMI 048583ii = QM 6874 = WB 2154 | EF669947 | EF669806 | EF669875 | EF669735 |
|  | *A. siamensis* | ^HT^ KUFC 6349 | N/A | AB646989 | N/A | N/A |
|  | *A. solicola* | ^HT^ NRRL 35723 | EU220279 | EU220283 | EU220284 | N/A |
|  | *A. spathulatus* | ^T^ NRRL 20549 = ATCC 64222 = NHL 2948 | EF669943 | EF669803 | EF669872 | EF669731 |
|  | *A. spinosus* | ^LT^ NRRL 5034 | EF669988 | EF669844 | EF669914 | EF669775 |
|  | *A. stramenius* | ^NT^ CBS 498.65 = NRRL 4652 = ATCC 16895 = IFO 9611 = IMI 172293 = QM 8900 = WB 4652 | EF669984 | EF669840 | EF669910 | EF669771 |
|  | *A. takakii* | ^HT^ CBM-FA-884 | N/A | AB787221 | AB787566 | N/A |
|  | *A. tatenoi* | ^HT^ CBS 407.93 = CBM FA 0022 | MH862426 | DQ114130 | DQ114139 | N/A |
|  | *A. turcosus* | ^HT^ KACC 42091 = IBT 27921 | N/A | DQ534143 | DQ534148 | HF545310 |
|  | *A. udagawae* (*N. udagawae*) | ^HT^ CBM FA-0702 | AB250781 | AF132226 | AB748566 | N/A |
|  | *A. unilateralis* | ^NT^ NRRL 577 = CBS 126.56 = ATCC 16902 = IFO 8136 = IMI 062876 = QM 8163 = WB 4366 = WB 4779 | EF669997 | EF669852 | EF669923 | EF669784 |
|  | *A. wyomingensis* | ^HT^ CCF 4417 = CMF ISB 2494 | HG324081 | HF933359 | HF933397 | HF937378 |
| *Nigri* | *A. acidus* | ^T^ CBS 564.65 | AJ280009 | AY585533 | AY585533 | N/A |
|  | *A. aculeatinus* | ^T^ CBS 121060 = IBT 29077 | EU159211 | EU159220 | EU159241 | HF559233 |
|  | *A. aculeatus* | ^T^ CBS 172.66 = NRRL 5094 = IMI 211388 = ATCC 16872 = WB 5094 | EF661221 | HE577806 | EF661148 | EF661046 |
|  | *A. aculeatus* | F-719 | HE578071 | HE577810 | HE578093 |  |
|  | *A. awamori* | ^NT^ CBS 557.65 = ITEM 4509 = ATCC 16877 = IMI 211394 = IOC 230 = WB 4948 | AM087614 | AY820001 | AJ964874 | N/A |
|  | *A. brasiliensis* | ^HT^ CBS 101740 = IMI 381727 = IBT 21946 | FJ629321 | FJ629272 | FN594543 | KY006765 load again |
|  | *A. brunneoviolaceus* | ^T^ CBS 621.78 = NRRL 4912 = IMI 312981 = WB 4912 | AJ280003 | EF661105 | EF661147 | EF661045 |
|  | *A. brunneoviolaceus* | ITEM 14784 | N/A | HE984411 | HE984426 | HE984374 |
|  | *A. brunneoviolaceus* | ITEM 14785 | N/A | N/A | HE984427 | HE984375 |
|  | *A. brunneoviolaceus* | CCF 108 | FR727129 | FR775311 | HE608868 | N/A |
|  | *A. brunneoviolaceus* | IHEM 4062 | MH613091 | HE818085 | HE818079 | N/A |
|  | *A. brunneoviolaceus* | CBS 119.49 = ITEM 7037 | FJ491679 | FJ491689 | FJ491701 | N/A |
|  | *A. carbonarius* | ^NT^ CBS 111.26 = NRRL 369 = ATCC 1025 = IMI 016136 = LSHB Ac11 = NCTC 1325 = NRRL 1987 = QM 331 = WB 369 | EF661204 | EF661099 | EF661167 | EF661068 |
|  | *A. coreanus* | ^HT^ CBS 117059 = KACC 41659 = NRRL 35590 | JN943570 | AY870758 | AY870718 | N/A |
|  | *A. costaricaensis* | ^HT^ CBS 115574 = IBT 23401 = CECT 20579 = ITEM 7555 | DQ900602 | FJ629277 | FN594545 | HE984361 |
|  | *A. ellipticus* | ^T^ CBS 482.65 = NRRL 5120 = ATCC 16876 = IMI 172283 = QM 8886 = WB 5120 | EF661194 | EF661122 | EF661170 | EF661051 |
|  | *A. eucalypticola* | ^HT^ CBS 122712 = IBT 29274 | EU482439 | EU482435 | EU482433 | N/A |
|  | *A. fijiensis* | ^HT^ CBS 313.89 = IBT 13989 | FJ491680 | FJ491688 | FJ491695 | N/A |
|  | *A. floridensis* | ^HT^ NRRL 62478 = ITEM 14783 | N/A | HE984412 | HE984429 | HE984376 |
|  | *A. foetidus* | ^NT^ CBS 121.28 = NRRL 4784 = IFO 4031 = IMI 104687 = WB 4784 | FJ491683 | FJ491690 | FJ491694 | N/A |
|  | *A. heteromorphus* | ^NT^ CBS 117.55 = NRRL 4747 = ATCC 12064 = IMI 172288 = QM 6954 = WB 4747 | EU821305 | EF661103 | EF661169 | EF661050 |
|  | *A. homomorphus* | ^HT^ CBS 101889 = CECT 20580 | EF166063 | AY820015 | FN594549 | N/A |
|  | ***A. hydei*** | **KUMCC 18-0196** | **MT152332** | **MT161679** | **MT178247** | **MT384370** |
|  | *A. ibericus* | ^HT^ ITEM 4776 = IMI 391429 | NR_119514 | AM419748 | AJ971805 | N/A |
|  | *A. indologenus* | ^HT^ CBS 114.80 = IBT 3679 = ITEM 7038 | AJ280005 | AY585539 | AM419750 | HE984366 |
|  | *A. japonicus* | ^NT^ CBS 114.51 | AJ279985 | HE577804 | FN594551 | N/A |
|  | *A. labruscus* | ^HT^ CCT 7800 HT = IBT 33586 = ITAL 22.223 | KU708544 | KT986014 | KT986008 | N/A |
|  | *A. lacticoffeatus* | ^HT^ CBS 101883 = IBT 22031 = ITEM 7559 | FJ629336 | AY819998 | EU163270 | HE984367 |
|  | *A. luchuensis* | ^T^ CBS 205.80 = IFO 428 |  |  |  |  |
|  | *A. neoniger* | ^HT^ CBS 115656 = NRRL 62634 | FJ491682 | FJ491691 | FJ491700 | KC796429 |
|  | *A. niger* | ^NT^ CBS 554.65 = NRRL 326 = ATCC 16888 = IFO 33023 = IHEM 3415 = IMI 50566 = IMI 050566ii = JCM 10254 = QM 9270 = QM 9946 = Thom 2766 = WB 326 | EF661186 | EF661089 | EF661154 | EF661058 |
|  | *A. piperis* | ^HT^ CBS 112811 = IBT 24630 = IBT 26239 = NRRL 62631 | EU821316 | FJ629303 | EU163267 | KC796427 |
|  | *A. sclerotiicarbonarius* | ^HT^ CBS 121057 = IBT 28362 | EU159216 | EU159229 | EU159235 | N/A |
|  | *A. saccharolyticus* | ^HT^ CBS 127449 = IBT 28509 = IBT 30881 | HM853552 | HM853553 | HM853554 | HF559235 |
|  | *A. sclerotioniger* | ^HT^ CBS 115572 = IBT 22905 = ITEM 7560 | DQ900606 | AY819996 | FN594557 | HE984369 |
|  | *A. trinidadensis* | ^HT^ NRRL 62479 = ITEM 14821 | N/A | HE984420 | HE984434 | HE984379 |
|  | *A. tubingensis* | ^T^ NRRL 4875 = QM 8904 = WB 4875 | EF661193 | EF661086 | EF661151 | EF661055 |
|  | *A. uvarum* | ^HT^ CBS 121591 = IBT 26606 = IMI 388523 = ITEM 4834 | AM745757 | AM745751 | AM745755 | HE984370 |
|  | *A. vadensis* | ^T^ CBS 113365 = CECT 20584 = IMI 313493 = ITEM 7561 | AY585549 | AY585531 | FN594560 | HE984371 |
|  | *A. violaceofuscus* | ^T^ CBS 102.23 | FJ491677 | FJ491686 | FJ491697 | HF559234 |
|  | *A. welwitschiae* | ^ET^ CBS 139.54 | MH857271 | FJ629291 | KC480196 | N/A |
|  | *Dichotomocladium elegans* | ^T^ CBS 714.74 = ATCC 32874 = IMI 11271 = NRRL 2664 = RSA 601 | JN205839 | N/A | N/A | N/A |
|  | ***Fusarium* sp.** | **KUMCC 18-0205** | **MT152341** | **N/A** | **N/A** | **N/A** |
|  | *Gongronella brasiliensis* | ^HT^ URM 7487 | KY114930 | N/A | N/A | N/A |
|  | *G. brasiliensis* | URM 7488 | KY114931 | N/A | N/A | N/A |
|  | *G. butleri* | ^T^ CBS 216.58 (type of *Mucor vesiculosus*) | JN206285 | N/A | N/A | N/A |
|  | *G. butleri* | CBS 415.67 | JN206288 | N/A | N/A | N/A |
|  | *G. butleri* | CBS 102.44 | JN206284 | N/A | N/A | N/A |
|  | ***G. butleri*** | **KUMCC 18-0201** | **MT152333** | **N/A** | **N/A** | **N/A** |
|  | *G. guangdongensis* | ^HT^ LC 1994 = CGMCC 3.15212 | KC462739 | N/A | N/A | N/A |
|  | *G. guangdongensis* | LC 1995 | KC462740 | N/A | N/A | N/A |
|  | ***G. hydei*** | **^HT^ KUMCC 18-0198** | **MT152334** | **N/A** | **N/A** | **N/A** |
|  | ***G. hydei*** | **KUMCC 18-0204** | **MT152335** | **N/A** | **N/A** | **N/A** |
|  | *G. koreana* | ^HT^ EML-TS2Bp | KP636529 | N/A | N/A | N/A |
|  | *G. lacrispora* | ^HT^ CBS 244.62 = ATCC 4412 = DSM 1169 = NRRL 2643 | MH858146 | N/A | N/A | N/A |
|  | *G. orasabula* | ^HT^ EML-QF 12-1 | KT936269 | N/A | N/A | N/A |
|  | *G. orasabula* | EML-QF 12-2 | KT936270 | N/A | N/A | N/A |
|  | *G. sichuanensis* | ^HT^ CGMCC 3.19651 = GZUIFR H25.4.1 | MK813373 | N/A | N/A | N/A |
|  | *G. sichuanensis* | CGMCC 3.19652 | MK813374 | N/A | N/A | N/A |
|  | *G. sichuanensis* | CGMCC 3.19653 | MK813375 | N/A | N/A | N/A |
|  | *Lichtheimia corymbifera* | ^NT^ CBS 429. 75 (neotype of *Mucor corymbifer*) | GQ342878 | N/A | N/A | N/A |
| *Aspergilloides* | *Penicillium glabrum* (outgroup) | ^NT^ CBS 125543 = IBT 22658 = IMI 91944 = DAOM 227653 = DTO 076-G8 | GU981567 | GU981619 | KM089152 | JF417447 |
|  | *P. saturniforme* (outgroup) | ^HT^ CBS 122276 = AS 3.6886 | EU644081 | EU644080 | EU644062 | JN121439 |
| *Lanata*-*Divaricata* | *P. abidjanum* | ^HT^ CBS 246.67 = ATCC 18385 = FRR 1156 = IMI 136244 | GU981582 | GU981650 | KF296383 | JN121469 |
|  | *P. alagoense* | ^HT^ URM 8086 | MK804502 | MK802333 | MK802336 | MK802338 |
|  | *P. amphipolaria* | ^HT^ DAOMC 250551 = CBS 140997 = W 284 = KAS 2555 | KT887872 | KT887833 | KT887794 | N/A |
|  | *P. annulatum* | ^HT^ = CBS 135126 = CV 37 = DTO 180-G7 = KAS 4119 | JX091426 | JX091514 | JX141545 | KF296410 |
|  | *P. araracuarense* | ^HT^ CBS 113149 = IBT 23247 | GU981597 | GU981642 | KF296373 | KF296414 |
|  | *P. bissettii* | ^HT^ DAOM 167011 = CBS 140972 = KAS 1951 | KT887845 | KT887806 | KT887767 | N/A |
|  | *P. brasilianum* | ^HT^ CBS 253.55 = ATCC 12072 = FRR 3466 = QM 6947 | GU981577 | GU981629 | AB667857 | KF296420 |
|  | *P. brefeldianum* | ^T^ CBS 235.81 = NRRL 710 = FRR 710 = IFO 31731 = IMI 216896 | AF033435 | GU981623 | EU021683 | KF296421 |
|  | *P. camponotum* | ^HT^ DAOMC 250557 = CBS 140982 = NBBR-2-1 = W 471 = KAS 2177 | KT887855 | KT887816 | KT887777 | N/A |
|  | *P. caperatum* | ^T^ CBS 443.75 = ATCC 28046 = DSM 2209 = NHL 6465 | KC411761 | GU981660 | KF296392 | KF296422 |
|  | *P. cataractarum* | ^HT^ DAOM 250534 = CBS 140974 = W 4 = KAS 2145 | KT887847 | KT887808 | KT887769 | N/A |
|  | *P. cluniae* | ^T^ CBS 326.89 | KF296406 | KF296471 | KF296402 | KF296424 |
|  | *P. coeruleum* | ^T^ CBS 141.45 = NCTC 6595 | GU981606 | GU981655 | KF296393 | KF296425 |
|  | *P. cremeogriseum* | ^NT^ CBS 223.66 | GU981586 | GU981624 | KF296403 | KF296426 |
|  | *P. curticaule* | ^HT^ CBS 135127 = CV 2842 = DTO 180-D3 = DAOM 241159 | FJ231021 | JX091526 | JX141536 | KF296417 |
|  | *P. daleae* | ^T^ CBS 211.28 = ATCC 10435 = DSM 2449 = FRR 2025 = IFO 6087 = IFO 9072 = MUCL 29234 = NRRL 2025 | GU981583 | GU981649 | KF296385 | KF296427 |
|  | *P. echinulonalgiovense* | ^HT^ CBS 328.59 = ATCC 18314 = FAT 907 = FRR 638 = IFO 6229 = IMI 68213 = QM 7301 | GU981587 | GU981631 | KX961269 | KX961301 |
|  | *P. ehrlichii* | ^HT^ CBS 324.48 = NRRL 708 | AF033432 | KF296464 | KF296395 | KF296428 |
|  | *P. elleniae* | ^HT^ CBS 118135 = IBT 23229 | GU981612 | GU981663 | KF296389 | KF296429 |
|  | *P. excelsum* | ^HT^ ITAL 7572 = CCT 7772 = IBT 31516 | KR815341 | KP691061 | KR815342 | N/A |
|  | *P. flaviroseum* | ^HT^ CGMCC 3.18805 = CBS 144479 | KY495032 | KY495141 | KY494972 | KY495083 |
|  | *P. fructuariae-cellae* | ^HT^ ITEM 18276 | MK039434 | KU554679 | MK045337 | MK520927 |
|  | *P. glaucoroseum* | ^ET^ NRRL 908 = CBS 138908 | KF296407 | KF296469 | KF296400 | KF296430 |
|  | *P. globosum* | ^HT^ CGMCC 3.18800 = CBS 144639 = NN 072354 | KY495014 | KY495123 | KY494954 | KY495067 |
|  | *P. griseoflavum* | ^HT^ CGMCC 3.18799 = CBS 144525 = NN 072331 | KY495011 | KY495120 | KY494951 | KY495064 |
|  | *P. griseopurpureum* | ^NT^ CBS 406.65 = IMI 96157 | KF296408 | KF296467 | KF296384 | KF296431 |
|  | *P. guangxiense* | ^HT^ CGMCC 3.18793 = CBS 144526 | KY494986 | KY495095 | KY494926 | N/A |
|  | *P. guaibinense* | ^HT^ CCDCA 11512 = 23EM8 | MH674389 | MH674391 | MH674393 | N/A |
|  | *P. guaibinense* | KUMCC 18-0199 | MT152336 | MT161680 | MT178248 | MT384371 |
|  | *P. guaibinense* | ^HT^ CGMCC 3.18797 = CBS 144505 = NN 072318 (ex-type of *P. austrosinense*) | KY495007 | KY495116 | KY494947 | KY495061 |
|  | *P. hainanense* | ^HT^ CGMCC 3.18798 = CBS 144527 | KY495009 | KY495118 | KY494949 | N/A |
|  | ***P. soli*** | **KUMCC 18-0202** | **MT152337** | **MT161681** | **MT178249** | **MT384372** |
|  | *P. infrabuccalum* | ^HT^ DAOMC 250537 = CBS 140983 = NBSM-6-2 = W 475 = KAS 2181 | KT887856 | KT887817 | KT887778 | N/A |
|  | *P. janthinellum* | ^NT^ CBS 340.48 = ATCC 10455 = IMI 40238 | GU981585 | GU981625 | KF296401 | JN121497 |
|  | *P. janthinellum* | NN 072417 | KY495025 | KY495134 | KY494965 | KY495076 |
|  | ***P. janthinellum*** | **KUMCC 18-0206** | **MT152338** | **MT161682** | **MT178250** | **MT384373** |
|  | *P. javanicum* | ^HT^ CBS 341.48 = NRRL 707 | GU981613 | GU981657 | KF296387 | JN121498 |
|  | *P. jianfenglingense* | ^HT^ CGMCC 3.18802 = CBS 144640 = NN 072384 | KY495016 | KY495125 | KY494956 | KY495069 |
|  | *P. koreense* | ^HT^ KACC 47721 | KJ801939 | KM000846 | N/A | N/A |
|  | *P. laevigatum* | ^HT^ CGMCC 3.18801 = CBS 144481 | KY495015 | KY495124 | KY494955 | KY495068 |
|  | *P. levitum* | ^NT^ CBS 345.48 = ATCC 10464 = IFO 6101 = IMI 39735 | GU981607 | GU981654 | KF296394 | KF296432 |
|  | *P. limosum* | ^HT^ CBS 339.97 = NEI 5220 | GU981568 | GU981621 | KF296398 | KF296433 |
|  | *P. lineolatum* | ^HT^ CBS 188.77 = NHL 2776 | GU981579 | GU981620 | KF296397 | KF296434 |
|  | *P. ludwigii* | ^T^ CBS 417.68 = FRR 559 | KF296409 | KF296468 | KF296404 | KF296435 |
|  | *P. malacosphaerulum* | ^HT^ CBS 135121 = CV 2855 = DTO 180-E6 = DAOM 241161 | FJ231026 | JX091524 | JX141542 | KF296438 |
|  | *P. mariae-crucis* | ^T^ CBS 271.83 = IMI 256075 = CECT 2742 | GU981593 | GU981630 | KF296374 | KF296439 |
|  | *P. meloforme* | ^HT^ CBS 445.74 = ATCC 28049 = IMI 216903 = NHL 6468 | KC411762 | GU981656 | KF296396 | KF296440 |
|  | *P. ochrochloron* | ^NT^ CBS 357.48 = ATCC 10540 = IMI 039806 = NRRL 926 = QM 7604 | GU981604 | GU981672 | KF296378 | KF296445 |
|  | *P. onobense* | ^NT^ CBS 174.81 = ATCC 42225 = IJFM 3026 = VKM F-2183 | GU981575 | GU981627 | KF296371 | KF296447 |
|  | *P. ortum* | ^HT^ CBS 135669 = CV 0102 = DTO 180-I9 = KAS 3962 | JX091427 | JX091520 | JX141551 | KF296443 |
|  | *P. ortum* | ^HT^ CBS 135670 = CV 0391 = DTO 181-F5 = KAS 4123 | JX091428 | JX091521 | JX141552 | KF296444 |
|  | *P. oxalicum* | ^NT^ CBS 219.30 = ATCC 1126 = FRR 787 = IMI 192332 = MUCL 29047 = NRRL 787 = QM 7606 | AF033438 | KF296462 | KF296367 | JN121456 |
|  | *P. panissanguineum* | ^HT^ DAOMC 250562 = CBS 140989 = W 93 = KAS 2209 | KT887862 | KT887823 | KT887784 | N/A |
|  | *P. paraherquei* | ^NT^ CBS 338.59 = ATCC 22354 = ATCC 46903 = FAT 964 = FRR 3454 = IFO 6234 = IMI 68220 = NRRL 3454 | AF178511 | KF296465 | KF296372 | KF296449 |
|  | *P. pedernalense* | ^HT^ CBS 140770 = CECT 20949 | KU255398 | KU255396 | N/A | N/A |
|  | *P. penarojense* | ^HT^ CBS 113178 = IBT 23262 | GU981570 | GU981646 | KF296381 | KF296450 |
|  | *P. piscarium* | ^NT^ CBS 362.48 = ATCC 10482 = FRR 1075 = IFO 8111 = IMI 40032 = NRRL 1075 = VKM F-1823 | GU981600 | GU981668 | KF296379 | KF296451 |
|  | *P. pulvillorum* | ^NT^ CBS 280.39 = IFO 7763 = NRRL 2026 | AF178517 | GU981670 | KF296377 | KF296452 |
|  | *P. raperi* | ^NT^ CBS 281.58 = NRRL 2674 | AF033433 | GU981622 | KF296399 | KF296453 |
|  | *P. reticulisporum* | ^HT^ CBS 122.68 = ATCC 18566 = IFO 9024 = IMI 136700 = NHL 6105 = NRRL 3447 | AF033437 | N/A | KF296391 | KF296454 |
|  | *P. rolfsii* | ^NT^ CBS 368.48 = ATCC 10491 = FRR 1078 = IFO 7735 = IMI 40029 = MUCL 29229 = NRRL 1078 = QM 1961 | JN617705 | GU981667 | KF296375 | KF296455 |
|  | *P. rubriannulatum* | ^HT^ CGMCC 3.18804 = CBS 144641 = NN072456 | KY495029 | KY495138 | KY494969 | KY495080 |
|  | *P. setosum* | ^HT^ CBS 144865 = MCC 1370 = NCFT NO 8222.16 = AMH-9974 | KT852579 | MF184995 | MH105905 | MH016196 |
|  | *P. simplicissimum* | ^T^ CBS 372.48= ATCC 10495 = FRR 902 = IFO 5762 = IMI 039816 = QM 1939 | GU981588 | GU981632 | KF296368 | JN121507 |
|  | *P. singorense* | ^HT^ CBS 138214 = DTO 133-C6 | KJ775674 | KJ775167 | KJ775403 | N/A |
|  | *P. skrjabinii* | ^NT^ CBS 439.75 = NRRL 13055 = FRR 1945 = IMI 196528 = VKM F-1940 | GU981576 | GU981626 | KF296370 | EU427252 |
|  | *P. soliforme* | ^HT^ CGMCC 3.18806 = CBS 144482 = NN072519 | KY495038 | KY495147 | KY494978 | N/A |
|  | *P. spinuliferum* | ^HT^ CGMCC 3.18807 = CBS 144483 = NN072545 | KY495040 | KY495149 | KY494980 | KY495090 |
|  | *P. subrubescens* | ^HT^ CBS 132785 = DTO 188-D6 = FBCC 1632 = IBT 31985 | KC346350 | KC346327 | KC346330 | KC346306 |
|  | *P. svalbardense* | ^HT^ CBS 122416 = IBT 23856 | GU981603 | N/A | KC346338 | KF296457 |
|  | *P. terrarumae* | ^HT^ HGUP 2025 = CBS 131811 = DTO 174-H2 | KX650288 | KX650295 | N/A | N/A |
|  | *P. tanzanicum* | ^HT^ DAOMC 250514 = CBS 140968 = 50.118 = KAS 1946 | KT887841 | KT887802 | KT887763 | N/A |
|  | *P. vanderhammenii* | ^HT^ CBS 126216 = IBT 23203 | GU981574 | GU981647 | KF296382 | KF296458 |
|  | *P. vasconiae* | ^NT^ CBS 339.79 = ATCC 42224 = IJFM 3008 | GU981599 | GU981653 | KF296386 | KF296459 |
|  | *P. viridissimum* | ^HT^ CGMCC 3.18796 = CBS 144484 = NN070281 | KY495004 | KY495113 | KY494944 | KY495059 |
|  | *P. wandoense* | ^HT^ CNUFC-WT31-1 | N/A | MK080564 | MK080566 | MK080562 |
|  | *P. wotroi* | ^HT^ CBS 118171 = IBT 23253 | GU981591 | GU981637 | KF296369 | KF296460 |
|  | *P. yunnanense* | ^HT^ CGMCC 3.18794 = CBS 144485 | KY494990 | KY495099 | KY494930 | KY495048 |
|  | *P. zonatum* | ^HT^ CBS 992.72 = ATCC 24353 | GU981581 | GU981651 | KF296380 | KF296461 |
| *Helici* | *Talaromyces boninensis* | ^HT^ CBS 650.95 = IBT 17516 | JN899356 | KJ865721 | KJ885263 | KM023276 |
|  | *T. helicus* | ^T^ CBS 335.48 | JN899359 | KJ865725 | KJ885289 | KM023273 |
| *Talaromyces* | *T. aculeatus* | ^NT^ CBS 289.48 = ATCC 10409 = IMI 40588 = NRRL 2129 = NRRL A-1474 | KF741995 | KF741929 | KF741975 | KM023271 |
|  | *T. adpressus* | ^HT^ CGMCC 3.18211 = CBS 140620 = DTO 317-G4 | KU866657 | KU866844 | KU866741 | KU867001 |
|  | *T. alveolaris* | ^HT^ CBS 142379 = UTHSC DI16-147 = FMR 13963 | LT558969 | LT559086 | LT795596 | LT795597 |
|  | *T. amazonensis* | ^HT^ CBS 140373 = IBT 23215 = DTO 093-F9 | KX011509 | KX011490 | KX011502 | N/A |
|  | *T. amestolkiae* | ^HT^ CBS 132696 = DTO 179-F5 | JX315660 | JX315623 | KF741937 | JX315698 |
|  | *T. angelicae* | ^HT^ KACC 46611 | KF183638 | KF183640 | KJ885259 | KX961275 |
|  | *T. apiculatus* | ^HT^ CBS 312.59 = ATCC 18315 = FRR 635 = IMI 68239 | JN899375 | KF741916 | KF741950 | KM023287 |
|  | *T. argentinensis* | ^HT^ NRRL 28750 | MH793045 | MH792917 | MH792981 | MH793108 |
|  | *T. aspriconidius* | ^HT^ CBS 141835 = DTO 340-F8 | MN864274 | MN863343 | MN863320 | MN863332 |
|  | *T. aurantiacus* | ^HT^ CBS 314.59 = ATCC 13216 = IMI 99722 = NRRL 3398 = QM 7722 | JN899380 | KF741917 | KF741951 | KX961285 |
|  | *T. australis* | ^HT^ CBS 137102 = DTO 273-F5 = IBT 14256 = FRR 2005 | KF741991 | KF741922 | KF741971 | N/A |
|  | *T. beijingensis* | ^HT^ CGMCC 3.18200 = CBS 140617 = DTO 317-D8 | KU866649 | KU866837 | KU866733 | KU866993 |
|  | *T. brevis* | ^HT^ CBS 141833 = DTO 349-E7 | MN864269 | MN863338 | MN863315 | MN863328 |
|  | *T. brevis* | DTO 307-C1 | MN864270 | MN863339 | MN863316 | MN863329 |
|  | *T. brevis* | CBS 118436 = DTO 004-D8 | MN864271 | MN863340 | MN863317 | MN863330 |
|  | *T. calidicanius* | ^HT^ CBS 112002 | JN899319 | HQ156944 | KF741934 | KM023311 |
|  | *T. californicus* | ^HT^ NRRL 58168 | MH793056 | MH792928 | MH792992 | MH793119 |
|  | *T. cnidii* | ^HT^ KACC 46617 | KF183639 | KF183641 | KJ885266 | KM023299 |
|  | *T. coprophilus* | ^HT^ FMR 15199 = CBS 142756 | LT899794 | LT898319 | LT899776 | LT899812 |
|  | *T.cucurbitiradicus* | ^HT^ ACCC 39155 | KY053254 | KY053228 | KY053246 | N/A |
|  | *T. derxii* | ^HT^ CBS 412.89 = NHL 2981 | JN899327 | JX494306 | KF741959 | KM023282 |
|  | *T. dimorphus* | ^HT^ AS3.15692 = NN072337 | KY007095 | KY007111 | KY007103 | KY112593 |
|  | *T. domesticus* | ^HT^ NRRL 58121 | MH793055 | MH792927 | MH792991 | MH793118 |
|  | *T. duclauxii* | ^AUT^ CBS 322.48 = ATCC 10439 = IMI 40044 = MUCL 28672 = MUCL 29094 = MUCL 29212 = NRRL 1030 | JN899342 | JX091384 | KF741955 | JN121491 |
|  | *T. euchlorocarpius* | ^HT^ DTO 176-I3 = PF 1203 = DTO 176-I4 | AB176617 | KJ865733 | KJ885271 | KM023303 |
|  | *T. flavovirens* | ^ET^ CBS 102801 = IBT 27044 | JN899392 | JX091376 | KF741933 | KX961283 |
|  | *T. flavus* | ^NT^ CBS 310.38 = NRRL 2098 | JN899360 | JX494302 | KF741949 | JF417426 |
|  | *T. francoae* | ^HT^ CBS 113134 = IBT 23221 = DTO 056-D9 | KX011510 | KX011489 | KX011501 | N/A |
|  | *T. funiculosus* | ^NT^ CBS 272.86 = IMI 193019 = FRR 1630 | JN899377 | JX091383 | KF741945 | KM023293 |
|  | *T. fuscoviridis* | ^HT^ CBS 193.69 = IBT 14846 = IBT 32646 | KF741979 | KF741912 | KF741942 | N/A |
|  | *T. fusiformis* | ^HT^ CGMCC 3.18210 = CBS 140637 = DTO 317-F4 | KU866656 | KU866843 | KU866740 | KU867000 |
|  | *T. galapagensis* | ^HT^ CBS 751.74 = IFO 31796 = NBRC 101902 | JN899358 | JX091388 | KF741966 | N/A |
|  | ***T. yunnanensis*** | **KUMCC 18-0208** | **MT152339** | **MT161683** | **MT178251** | **N/A** |
|  | *T. indigoticus* | ^HT^ CBS 100534 = IBT 17590 | JN899331 | JX494308 | KF741931 | KX961278 |
|  | *T. intermedius* | ^IT^ CBS 152.65 | JN899332 | JX091387 | KJ885290 | KX961282 |
|  | *T. kabodanensis* | ^HT^ CBS 139564 = DTO 204-F2 = CCTU 850 | KP851981 | KP851986 | KP851995 | N/A |
|  | *T. kendrickii* | ^HT^ CBS 136666 = DTO 273-F4 = IBT 13593 | KF741987 | KF741921 | KF741967 | N/A |
|  | *T. lentulus* | ^HT^ AS3.15689 = NN071323 | KY007088 | KY007104 | KY007096 | KY112586 |
|  | *T. liani* | ^T^ CBS 225.66 = ATCC 18325 = ATCC 18331 = IMI 98480 = NRRL 3380 = VKM F-301 | JN899395 | JX091380 | KJ885257 | KX961277 |
|  | *T. louisianensis* | ^HT^ NRRL 35823 | MH793052 | MH792924 | MH792988 | MH793115 |
|  | *T. macrosporus* | ^HT^ CBS 317.63 = FRR 404 = IMI 197478 | JN899333 | JX091382 | KF741952 | KM023292 |
|  | *T. mae* | ^HT^ AS3.15690 = NN071328 | KY007090 | KY007106 | KY007098 | KY112588 |
|  | *T. malicola* | ^HT^ NRRL 3724 | MH909513 | MH909406 | MH909459 | MH909567 |
|  | *T. mangshanicus* | ^HT^ CGMCC 3.18013 = HMAS 248733 | KX447531 | KX447530 | KX447528 | KX447527 |
|  | *T. marneffei* | ^T^ CBS 388.87 | JN899344 | JX091389 | KF741958 | KM023283 |
|  | *T. muroii* | ^HT^ CBS 756.96 = PF 1153 | JN899351 | KJ865727 | KJ885274 | KX961276 |
|  | *T. mycothecae* | ^HT^ URM 7622 = CBS 142494 | MF278326 | LT855561 | LT855564 | LT855567 |
|  | *T. neofusisporus* | ^HT^ AS3.15415 = CBS 139516 | KP765385 | KP765381 | KP765383 | N/A |
|  | *T. oumae-annae* | ^HT^ CBS 138208 = DTO 269-E8 | KJ775720 | KJ775213 | KJ775425 | KX961281 |
|  | *T. panamensis* | ^HT^ CBS 128.89 = IMI 297546 | JN899362 | HQ156948 | KF741936 | KM023284 |
|  | *T. paucisporus* | ^T^ CBM-FA-0944 | AB176603 | N/A | N/A | N/A |
|  | *T. pinophilus* | ^NT^ CBS 631.66 = ATCC 36839 = CECT 2809 = DSM 1944 = IAM 7013 =IMI 114933 | JN899382 | JX091381 | KF741964 | KM023291 |
|  | *T. pinophilus* | CBS 173.91 | KM066206 | KM066141 | N/A | N/A |
|  | *T. pinophilus* | CBS 235.94 | KM066204 | KM066145 | N/A | N/A |
|  | *T. pinophilus* | CBS 101709 | KM066205 | KM066142 | KM520391 | N/A |
|  | ***T.* *pinophilus*** | **KUMCC 18-0203** | **MT152340** | **MT161684** | **MT178252** | **MT384374** |
|  | *T. pratensis* | ^HT^ NRRL 62170 | MH793075 | MH792948 | MH793012 | MH793139 |
|  | *T. primulinus* | ^HT^ CBS 321.48 | JN899317 | JX494305 | KF741954 | KM023294 |
|  | *T. pseudofuniculosus* | ^HT^ FMR 15307 = CBS 143041 | LT899796 | LT898323 | LT899778 | LT899814 |
|  | *T. purgamentorum* | ^HT^ CBS 113145 = IBT 23220 = DTO 056-E1 | KX011504 | KX011487 | KX011500 | N/A |
|  | *T. purpureogenus* | ^NT^ CBS 286.36 = IMI 91926 | JN899372 | JX315639 | KF741947 | JX315709 |
|  | *T. qii* | ^HT^ AS3.15414 = CBS 139515 | KP765384 | KP765380 | KP765382 | N/A |
|  | *T. rapidus* | ^HT^ UTHSC DI16-148 = CBS 142382 = FMR 14293 | LT558970 | LT559087 | LT795600 | LT795601 |
|  | *T. ruber* | ^NT^ CBS 132704 = DTO 193-H6 = IBT 10703 | JX315662 | JX315629 | KF741938 | JX315700 |
|  | *T. rubicundus* | ^HT^ CBS 342.59 | JN899384 | JX494309 | KF741956 | KM023296 |
|  | *T. rufus* | ^HT^ CBS 141834 = DTO 349-D7 = CGMCC 3.13203 | MN864272 | MN863341 | MN863318 | MN863331 |
|  | *T. rufus* | DTO 274-C5 | MN864273 | MN863342 | MN863319 | N/A |
|  | *T. sayulitensis* | ^HT^ CBS 138204 = DTO 245-H1 | KJ775713 | KJ775206 | KJ775422 | N/A |
|  | *T. siamensis* | ^NT^ CBS 475.88 = IMI 323204 | JN899385 | JX091379 | KF741960 | KM023279 |
|  | *T. soli* | ^HT^ NRRL 62165 | MH793074 | MH792947 | MH793011 | MH793138 |
|  | *T. stellenboschiensis* | ^HT^ CBS 135665 = DTO 181-A2 = DAOM 241021 = IBT 32631 | JX091471 | JX091605 | JX140683 | N/A |
|  | *T. stipitatus* | ^IT^ CBS 375.48 = ATCC 10500 = NRRL 1006 = IMI 039805 | JN899348 | KM111288 | KF741957 | KM023280 |
|  | *T. stollii* | ^HT^ CBS 408.93 | JX315674 | JX315633 | JX315646 | JX315712 |
|  | *T. thailandensis* | ^HT^ CBS 133147 = KUFC 3399 | JX898041 | JX494294 | KF741940 | KM023307 |
|  | *T. tumuli* | ^HT^ NRRL 62151 | MH793071 | MH792944 | MH793008 | MH793135 |
|  | *T. veerkampii* | ^HT^ CBS 500.78 = IBT 14845 = IBT 32648 | KF741984 | KF741918 | KF741961 | N/A |
|  | *T. verruculosus* | ^NT^ NRRL 1050 = CBS 388.48 = ATCC 10513 = DSM 2263 = IMI 40039 | KF741994 | KF741928 | KF741974 | KM023306 |
|  | *T. verruculosus* | CBS 254.56 = IBT 5047 | KF741980 | KF741913 | KF741944 | N/A |
|  | *T. viridis* | ^IT^ CBS 114.72 = Warcup A 165/3 = Orr 3156 = NRRL 5575 = QM 9817 = ATCC 22467 | MH860406 | JX494310 | KF741935 | JN121430 |
|  | *T. viridulus* | ^HT^ CBS 252.87 = IMI 288716 = ATCC 56972 | JN899314 | JX091385 | KF741943 | JF417422 |
|  | *T. xishaensis* | ^HT^ CGMCC 3.17995 = HMAS 248732 | KU644580 | KU644581 | KU644582 | N/A |
| *Trachyspermi* | *T. trachyspermus* (outgroup) | ^HT^ CBS 373.48 = ATCC 10497 = IMI 040043 = NRRL 1028 | JN899354 | KF114803 | KJ885281 | JF417432 |
|  | *T. ucrainicus* (outgroup) | ^T^ CBS 162.67 = ATCC 22344 = FRR 3462 = NHL 6086 | JN899394 | KF114771 | KJ885282 | KM023289 |

**^a^**The newly generated sequences are in black bold. ^AUT^=authentic strain, ^ET^=ex-epitype, ^HT^=ex-holotype, ^IT^=ex-isotype, ^LT^=ex-lectotype, ^NT^=ex-neotype, ^T^=ex-type, ^R^=representative strain.

**TABLE S2** Phosphate solubilization of tricalcium phosphate (TCP) on solid and liquid Pikovskaya (PVK) media with pH values by 13 fungal strains and control.

| **Phosphate solubilization of TCP** | | **Day** | **Control** | **KUMCC** | | | | | | | | | | | |  |
| --- | --- | --- | --- | --- | --- | --- | --- | --- | --- | --- | --- | --- | --- | --- | --- | --- |
|  |  |  |  | **18-0196** | **18-0197** | **18-0198** | **18-0199** | **18-0200** | **18-0201** | **18-0202** | **18-0203** | **18-0204** | **18-0205** | **18-0206** | **18-0207** | **18-0208** |
| **Phosphate solubilization index (PSI) on solid agar plates (cm)** | | **1** | 0.00  ±0.00 | 2.08  ±0.40^a^ | 1.00  ±0.00^c^ | 1.00  ±0.00^c^ | 1.00  ±0.00^c^ | 1.00  ±0.00^c^ | 1.00  ±0.00^c^ | 1.00  ±0.00^c^ | 1.00  ±0.00^c^ | 1.00  ±0.00^c^ | 1.00  ±0.00^c^ | 1.38  ±0.33^b^ | 1.00  ±0.00^c^ | 1.00  ±0.00^c^ |
|  |  | **3** | 0.00  ±0.00 | 2.34  ±0.47^a^ | 1.40  ±0.35^e^ | 2.04  ±0.13^b^ | 1.94  ±0.14^bc^ | 1.40  ±0.20^e^ | 1.97  ±0.12^bc^ | 1.62  ±0.09^de^ | 2.05  ±0.00^b^ | 1.95  ±0.12^bc^ | 1.68  ±0.04^cd^ | 1.92  ±0.16^bc^ | 1.38  ±0.10^e^ | 1.83  ±0.30^bcd^ |
|  |  | **5** | 0.00  ±0.00 | 2.57  ±0.08^a^ | 1.63  ±0.20^d^ | 2.07  ±0.15^b^ | 1.91  ±0.10^b^ | 1.35  ±0.04^e^ | 1.96  ±0.12^b^ | 1.61  ±0.10^d^ | 1.98  ±0.02^b^ | 2.03  ±0.50^b^ | 1.70  ±0.95^cd^ | 1.88  ±0.13^bc^ | 1.56  ±0.03^d^ | 1.70  ±0.17^cd^ |
|  |  | **7** | 0.00  ±0.00 | 2.58  ±0.04^a^ | 1.10  ±0.17^f^ | 1.97  ±0.15^b^ | 1.80  ±0.09^bcd^ | 1.31  ±0.03^e^ | 1.88  ±0.18^bc^ | 1.59  ±0.08^d^ | 1.90  ±0.01^bc^ | 1.88  ±0.16^bc^ | 1.58  ±0.07^d^ | 1.80  ±0.16^bcd^ | 1.69  ±0.02^cd^ | 1.73  ±0.22^cd^ |
| **Phosphate solubilization in broth medium** | **Phosphate solubilization**  **(µg/mL)** | **1** | 203.33  ±13.20^e^ | 469.67  ±41.48^a^ | 304.00  ±44.24^cd^ | 208.33  ±42.15^de^ | 217.67  ±83.94^de^ | 401.00  ±51.26^ab^ | 225.67  ±55.43^de^ | 213.67  ±24.84^de^ | 262.67  ±22.74^de^ | 263.33±23.86^de^ | 309.67±24.34^cd^ | 233.00  ±108.89^de^ | 223.00±50.47^de^ | 376.67±43.47^bc^ |
|  |  | **3** | 208.00  ±3.46^f^ | 760.00  ±11.53^a^ | 335.33  ±35.73^c^ | 316.33  ±24.38^cd^ | 232.00  ±71.76^ef^ | 550.33  ±38.50^b^ | 245.00  ±31.00^def^ | 243.33  ±14.19^def^ | 301.00  ±15.52^cde^ | 275.33  ±31.50^cdef^ | 339.67±16.86^c^ | 266.33  ±96.8^cdef^ | 260.00  ±53.67^cdef^ | 523.00±57.30^b^ |
|  |  | **5** | 213.33  ±5.77^g^ | 1352.00  ±33.05^a^ | 627.00  ±26.06^d^ | 618.00  ±43.71^d^ | 553.00  ±34.51^d^ | 1020.67  ±118.12^b^ | 412.33  ±15.82^e^ | 292.33  ±55.90^fg^ | 437.00  ±17.52^e^ | 454.33  ±35.28^e^ | 719.33±37.58^c^ | 418.33  ±48.64^e^ | 374.33  ±80.37^ef^ | 724.00±31.24^c^ |
|  |  | **7** | 213.33  ±6.66^g^ | 1523.33  ±47.87^a^ | 673.33  ±265.57^d^ | 671.67  ±49.17^d^ | 541.67  ±109.70^d^ | 982.67  ±225.62^b^ | 417.33  ±22.00^e^ | 461.67  ±64.61^fg^ | 570.67  ±26.10^e^ | 465.00  ±33.60^e^ | 637.33±50.56^c^ | 501.33  ±31.39^e^ | 632.67  ±25.00^ef^ | 766.33±18.00^c^ |
|  | **pH of culture filtrate with TCP** | **1** | 7.00  ±0.01^h^ | 5.82±0.11^ab^ | 6.11±0.03^d^ | 6.34±0.03^f^ | 6.58±0.08^g^ | 5.79±0.05^a^ | 5.88±0.04^b^ | 6.09±0.03^cd^ | 6.23±0.03^e^ | 6.00±0.03^c^ | 6.12±0.03^d^ | 6.10±0.05^d^ | 6.08±0.04^cd^ | 6.10±0.04^d^ |
|  |  | **3** | 6.74  ±0.36^i^ | 3.89±0.14^a^ | 5.32±0.08^def^ | 5.83±0.14^gh^ | 5.39±0.05^ef^ | 4.96±0.19^bcd^ | 5.08±0.07^cde^ | 4.56±0.06^b^ | 5.83±0.01^gh^ | 4.81±0.071^bc^ | 5.58±0.07^fg^ | 6.04±0.06^h^ | 5.70±0.08^fgh^ | 4.60±0.02^b^ |
|  |  | **5** | 6.07  ±0.12^g^ | 3.66±0.23^a^ | 4.42±0.18^bc^ | 4.66±0.23^cde^ | 4.78±0.05^def^ | 4.57±0.24^bcd^ | 4.32±0.03^b^ | 4.76±0.02^def^ | 4.52±0.07^bcd^ | 4.71±0.013^def^ | 4.61±0.17^cde^ | 4.93±0.06^f^ | 4.84±0.08^ef^ | 4.54±0.05^bcd^ |
|  |  | **7** | 6.00  ±0.01^l^ | 3.33±0.01^a^ | 4.24±0.01^d^ | 4.64±0.01^h^ | 5.08±0.02^i^ | 5.19±0.03^j^ | 4.01±0.02^c^ | 4.26±0.01^d^ | 3.92±0.03^b^ | 4.53±0.01^g^ | 5.25±0.05^k^ | 4.44±0.05^f^ | 4.47±0.04^f^ | 4.36±0.03^e^ |

Values given are the mean ± standard deviation of the three replicates

Different letters indicate significant difference based on Duncan at 𝑝 < 0.05.

**TABLE S3** Details of 13 phosphate-solubilizing fungal strains used in this study.

| **Strain no** | **Identified name** | **Host/ Locality** |
| --- | --- | --- |
| KUMCC 18-0196 | *Aspergillus hydei* sp. nov. | Air under *Quercus variabilis* tree/ Kunming City, Yunnan Province, China |
| KUMCC 18-0197 | *A. fumigatus* | Rhizosphere soil of *Quercus rubra*/ Honghe County, Yunnan Province, China |
| KUMCC 18-0198 and  KUMCC 18-0204 | *Gongronella hydei* sp. nov. |
| KUMCC 18-0199 | *Penicillium guaibinense* |
| KUMCC 18-0200 and  KUMCC 18-0207 | *A. flavus* |
| KUMCC 18-0201 | *G. butleri* |
| KUMCC 18-0202 | *P. soli* sp. nov. |
| KUMCC 18-0203 | *Talaromyces pinophilus* |
| KUMCC 18-0205 | *Fusarium* sp. |
| KUMCC 18-0206 | *P. janthinellum* |
| KUMCC 18-0208 | *T. yunnanensis* sp. nov. |

**TABLE S4** Nucleotide comparison of ITS, BenA, CaM and RPB2 between strains of *Penicillium guaibinense* and *P. austrosinense*.

| **Taxa** | **Contrast** | **Fragment length (bp), Number of different base pairs, Difference (%)** | | | |
| --- | --- | --- | --- | --- | --- |
|  |  | **ITS** | **BenA** | **CaM** | **RPB2** |
| *Penicillium guaibinense* (23EM8)**^T^** | *P. austrosinense* (NN072318)**^T^** | 486, 2, 0.41 | 420, 1, 0.24 | 398, 4, 1.01 | - |
|  | *P. austrosinense* (NN072022) | 486, 2, 0.41 | 420, 2, 0.48 | 398, 2, 0.50 | - |
|  | *P. austrosinense* (NN072385) | 486, 2, 0.41 | 420, 1, 0.24 | 398, 4, 1.01 | - |
| *P. guaibinense* (23EM7) | *P. austrosinense* (NN072318)**^T^** | 440, 2, 0.45 | 421, 3, 0.71 | 366, 4, 1.09 | - |
|  | *P. austrosinense* (NN072022) | 440, 2, 0.45 | 421, 4, 0.95 | 366, 2, 0.55 | - |
|  | *P. austrosinense* (NN072385) | 440, 2, 0.45 | 421, 3, 0.71 | 366, 4, 1.09 | - |
| *P. guaibinense* KUMCC 18-0199 | *P. austrosinense* (NN072318)**^T^** | 510, 1, 0.20 | 420, 1, 0.24 | 398, 2, 0.50 | 755, 6, 0.79 |
|  | *P. austrosinense* (NN072022) | 510, 1, 0.20 | 420, 0, 0 | 398, 2, 0.50 | 728, 6, 0.82 |
|  | *P. austrosinense* (NN072385) | 510, 1, 0.20 | 420, 1, 0.24 | 398, 2, 0.50 | - |
|  | *P. guaibinense* (23EM8)**^T^** | 522, 1, 0.19 | 495, 2, 0.40 | 501, 2, 0.4 | - |
|  | *P. guaibinense* (23EM7) | 440, 1, 0.23 | 496, 4, 0.81 | 445, 2, 0.45 | - |

**TABLE S5** List of phosphate-solubilizing fungi.

| **Taxa** | **Strain numbers** | **Substrate/locality** | **Method for fungal identification** | **References** |
| --- | --- | --- | --- | --- |
| *Aspergillus floccosus* | SI-2URAgr and SI-14URAgr | Dark red soil/ Nishihara, Okinawa, Japan | Morphological studies and  BenA sequence data | Islam et al., 2019 |
| *A. niger* | strain-1 and strain-2 | Soils/ Ny-Ålesund, Spitsbergen,  Svalbard | Based on morphology | Mohan Singh et al., 2011 |
| *A. niger* | CS-1 | Rhizosphere soil from wheat field/ Taian, Shandong, China | Phylogenetic analysis based on ITS | Wang et al., 2018 |
| *A. niger* | ML-107 | Soil/ Punjab, Pakistan | Morphology | Nelofer et al., 2015 |
| *A. niger* | SI-10URAgr, SI-11URAgr and SI-12URAgr) | Red soils/ Kunigami, Okinawa, Japan | Morphological studies and  BenA and CaM sequence data | Islam et al., 2019 |
| *A. neoniger* | AspN-JX16 | Rhizosphere soil of  moso bamboo (*Phyllostachys edulis*)/ China | Morphology and phylogenetic analysis based on ITS, BenA and CaM sequencedata | Zhang et al., 2018 |
| *A. niveus* | SI-3URAgr | Dark red soil/ Nishihara, Okinawa, Japan | Morphological studies and  BenA and CaM sequence data | Islam et al., 2019 |
| *Eupenicillium parvum* (current name *Penicillium parvum*) | FIHB 539 | Soil at the tea (*Camellia sinensis*)  experimental farm/ Banuri, Palampur in Himachal Pradesh, India | Morphology and phylogenetic analysis based on ITS sequence data | Vyas et al., 2007 |
| *P. guanacastense* | JP-NJ2 | Rhizosphere soil of *Pinus massoniana*/ China | Based on ITS sequence data | Qiao et al., 2019 |
| *Talaromyces aurantiacus* | TalA-JX04 | Rhizosphere soil of  moso bamboo (*Phyllostachys edulis*)/ China | Morphology and phylogenetic analysis of the CaM sequence data | Zhang et al., 2018 |
